# Aggregation-induced emission of siloles

**DOI:** 10.1039/c5sc01946j

**Published:** 2015-07-14

**Authors:** Zujin Zhao, Bairong He, Ben Zhong Tang

**Affiliations:** a State Key Laboratory of Luminescent Materials and Devices , South China University of Technology , Guangzhou 510640 , China . Email: mszjzhao@scut.edu.cn; b Department of Chemistry , The Hong Kong University of Science and Technology , Clear Water Bay , Kowloon , Hong Kong , China . Email: tangbenz@ust.hk

## Abstract

Aggregation-induced emission (AIE) is a unique and significant photophysical phenomenon that differs greatly from the commonly acknowledged aggregation-caused emission quenching observed for many π-conjugated planar chromophores. The mechanistic decipherment of the AIE phenomenon is of high importance for the advance of new AIE systems and exploitation of their potential applications. Propeller-like 2,3,4,5-tetraphenylsiloles are archetypal AIE-active luminogens, and have been adopted as a core part in the design of numerous luminescent materials with diverse functionalities. In this review article, we elucidate the impacts of substituents on the AIE activity and shed light on the structure–property relationship of siloles, with the aim of promoting the judicious design of AIE-active functional materials in the future. Recent representative advances of new silole-based functional materials and their potential applications are reviewed as well.

## Abstract 2

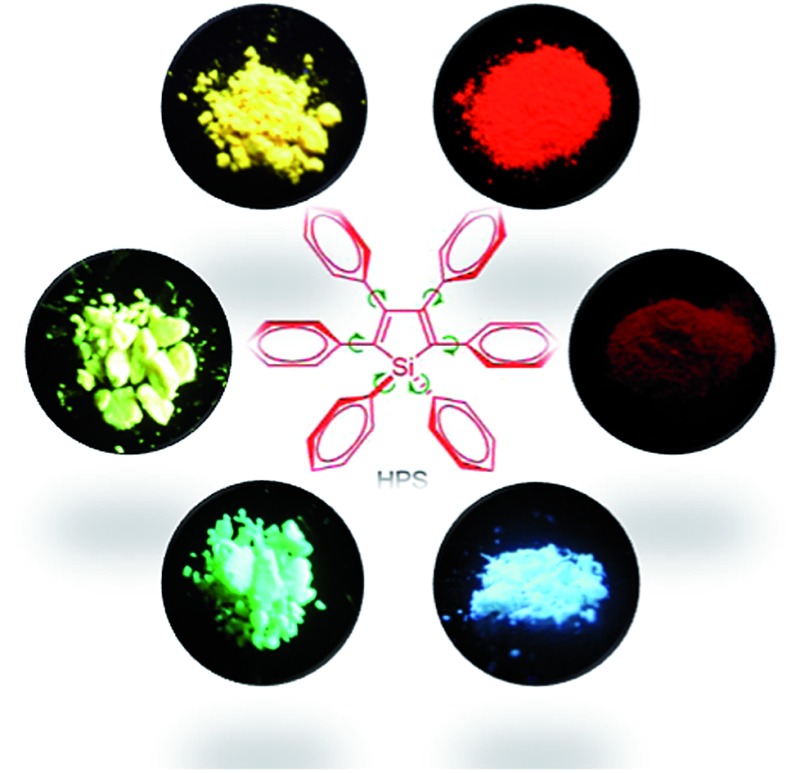
Recent advances in the structure–property relationship decipherment and luminescent functional materials development of AIE-active siloles are reviewed.

## 1. Introduction

Aggregation-induced emission (AIE),^1^ a novel light emission phenomenon, is receiving intense current research interest, due to its bright prospects in material science and biological technology. Luminogens with AIE characteristics are weakly fluorescent or non-fluorescent in the solution state, but can fluoresce strongly in the aggregated state, which differs greatly from the aggregation-caused quenching (ACQ) of light emission observed for many conventional chromophores that possess planar and well-conjugated structures. In most cases, conventional chromophores only show efficient light emission in a molecularly dispersive state and the agglomeration of the molecules should be strictly avoided, which has hampered severely the practical applications of many leading chromophores. The discovery of the fantastic AIE phenomenon furnishes a high possibility of conquering the ACQ problem and opens a new avenue towards efficient solid-state light emitters, bioprobes, chemosensors, and “smart” materials. Attracted by the intriguing AIE phenomenon, many research groups have enthusiastically embarked upon a search for new AIE luminogens, and the exploration of their practical utility.

Through systematic experimental measurements and theoretical calculations, it has been rationalized that the restriction of intramolecular motions (RIM), including rotation, vibration, stretching, *etc.*, is highly responsible for the AIE effect.^2^ In the solution state, active molecular motions serve as a relaxation channel for the excited state to decay nonradiatively, whereas in the aggregated state, these motions are suppressed greatly due to the spatial constraint, which blocks the nonradiative decay channel and promotes the radiative decay of the excited state.

**Scheme 1:**
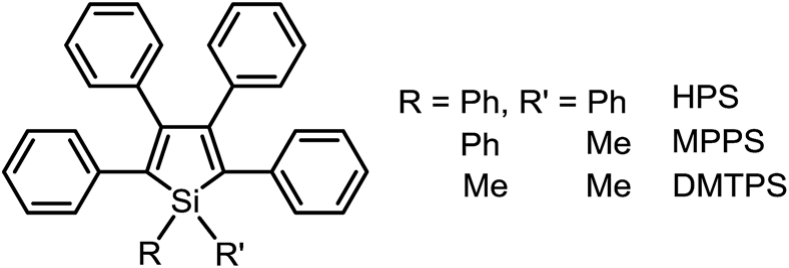
Chemical structures of 2,3,4,5-tetraphenylsilole derivatives.

**Fig. 1:**
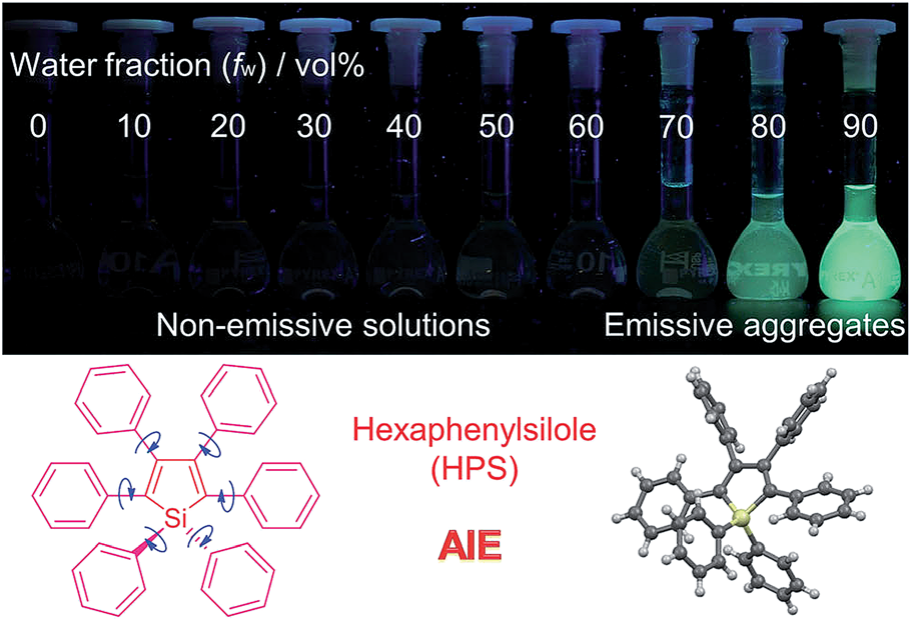
Fluorescence photographs of solutions and suspensions of hexaphenylsilole (HPS; 20 μM) in THF/water mixtures with different fractions of water. Reproduced with permission from ref. 2*c*. Copyright (2014) Wiley-VCH.

Based on the understanding of the AIE mechanism, various interesting AIE systems have been developed to date, including siloles,^1,3^ cyanostilbenes,^4^ tetraphenylethenes,^5,6^ anthracene derivatives,^7^ diphenyldibenzofulvenes,^8^ pyrrole and diketopyrrolopyrrole derivatives,^9,10^ phosphole and phosphindole oxides,^11^ pyrazine derivatives,^12^ ESIPT molecules,^13^ organoborons,^14^ *o*-carborane dyes,^15^ metal complexes,^16^ and other luminogenic molecules.^17^ Amongst these AIE-active luminogens, propeller-like siloles, such as 1,1-dimethyl-2,3,4,5-tetraphenylsilole (DMTPS), 1-methyl-1,2,3,4,5-pentaphenylsilole (MPPS) and 1,1,2,3,4,5-hexaphenylsilole (HPS) (Scheme 1), are the archetypes. They have high thermal stability and photostability, and can exist in acidic, neutral and weakly basic aqueous media. The propeller-like conformation endows these siloles with good solubility in common organic solvents, which facilitates purification and processing. Whereas they are almost nonfluorescent when molecularly dispersed in good solvents, they are good light emitters when fabricated into nanoaggregates (Fig. 1), crystals or solid films. In addition, siloles are also considered as a new kind of σ*–π* conjugated material with low-lying LUMO energy levels, deriving from the effective interaction between the σ* orbital of the exocyclic silicon–carbon bond and the π* orbital of the butadiene segment. Such a unique electronic structure imparts high electron affinity and fast electron mobility to siloles, and enables them to function as electron transporters in optoelectronic devices.^18^ Attracted by the AIE motif and interesting electronic features, numerous functional materials have been prepared by adopting silole as the luminescent core, which exhibit promising applications in organic light-emitting diodes,^19^ fluorescent bioprobes,^20^ chemosensors,^21,22^ *etc.*

Owing to the bright prospects of siloles in materials science and biotechnology, the development of new silole derivatives with altered emission colors and desired functionalities draws intense current research interest. Since the structure determines the properties, better understanding the structure–property relationship can guide the rational design of silole-based materials through molecular engineering endeavors in the future.^23^ A better understanding is conducive to a deeper mechanistic decipherment of the AIE phenomenon, and is meaningful for the advancement of new AIE systems and exploitation of their potential applications. Chemical modifications can be facilely carried out on the 1,2,3,4,5-positions of a silole ring, which offers abundant access to chemical diversity and specific properties. Although plenty of silole derivatives have been synthesized and utilized in various research fields, and some clues to the substitution effects on the photophysical properties and AIE activities of siloles have appeared, comprehensive and systematic studies on the structure–property relationships of AIE-active silole systems are rarely conducted. To depict a clear cut picture of the impacts of substituents on the optical properties of AIE-active siloles, in this review article, we will elaborate on typical siloles with different emission wavelengths and efficiencies. It is demonstrated that substituents at the 2,5-positions determine the emission wavelength of a silole to a large degree, while those at the 3,4-positions are crucial to keeping the AIE properties. Whereas groups at the 1-position hardly exert any effects on the emission of a silole in the solution state, their influence on the solid-state emission properties can't be neglected. After the discussion on the structure–property correlation, the recent advances of new siloles with representative applications in the fields of chemosensors, fluorescent bioprobes and bioimaging, chiral supramolecular self-assembly, and organic light-emitting diodes are presented.

## 2. Structure–property correlation

### 2.1. Siloles with nonfluorescent substituents

**Scheme 2:**
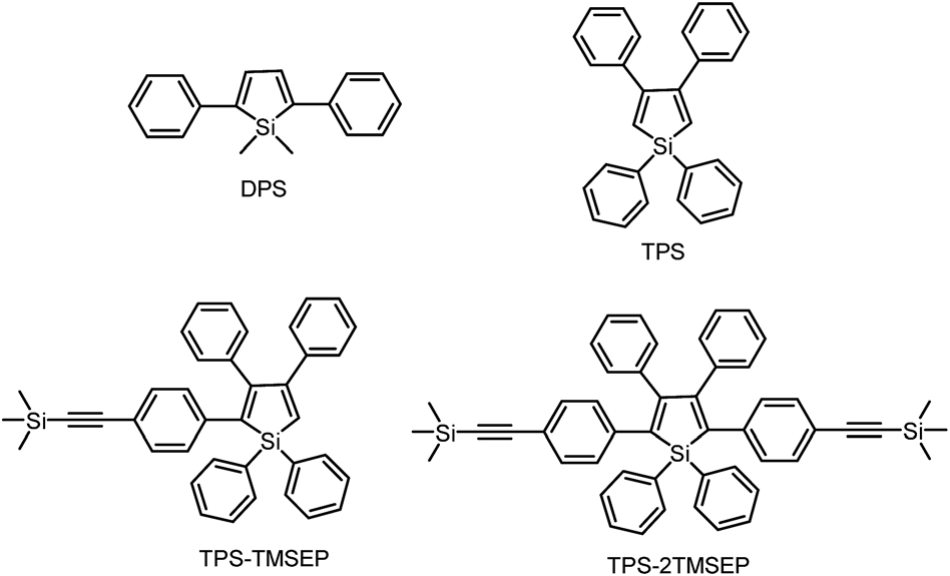
Chemical structures of the siloles DPS, TPS, TPS-TMSEP and TPS-2TMSEP.

1,1-Dimethyl-2,5-diphenylsilole (DPS) is a simple-structured silole derivative (Scheme 2). The molecule is not very sterically crowded because of the absence of substituents at the 3,4-positions, and thus, the phenyl rings at the 2,5-positions can effectively conjugate with the silole ring, allowing for a planar and relatively rigid molecular conformation. The energy consumed during the intramolecular rotation (IMR) process is reduced, enabling the molecule to fluoresce strongly in the solution state. Its emission peak is located at 463 nm in THF solution, with a fluorescence quantum yield (*Φ*_F_) of 29%.^24^ However, 1,1,3,4-tetraphenylsilole (TPS), with two phenyl rings at the 3,4-positions rather than 2,5-positions, possesses a twisted and poorly conjugated conformation. TPS has an absorption maximum at 288 nm, being blue-shifted by 88 nm in comparison to that of DPS (376 nm). TPS shows very weak emission peaks at ∼392 nm in solution and when aggregated, due to the limited conjugation within the molecule. With the addition of 4-((trimethylsilyl)ethynyl)phenyl groups at the 2,5-positions of the silole ring in TPS, the dihedral angles at the 3,4-positions become larger in order to reduce the steric repulsion. Meanwhile, the conjugation is extended through to the 2,5-positions, resulting in greatly red-shifted absorption and emission spectra. TPS-TMSEP and TPS-2TMSEP show no emissions in the solution state, but they exhibit strong emissions peaking at 491 and 517 nm, with high *Φ*_F_ values of ∼55% and ∼91% in the solid state, respectively, revealing that they are AIE-active.

By comparing the optical properties and crystal structures of these silole molecules, it becomes clear that substituents at the 3,4-positions are connected in a twisted manner to the silole ring to reduce steric congestion, regardless of whether there are substituents at the 2,5-positions. They are also involved in steric repulsion with the substituents at the 2,5-positions. Without phenyl rings at the 3,4-positions, the phenyl rings at the 2,5-positions can conjugate with the silole ring much better, resulting in a more planar and rigid conformation.

**Scheme 3:**
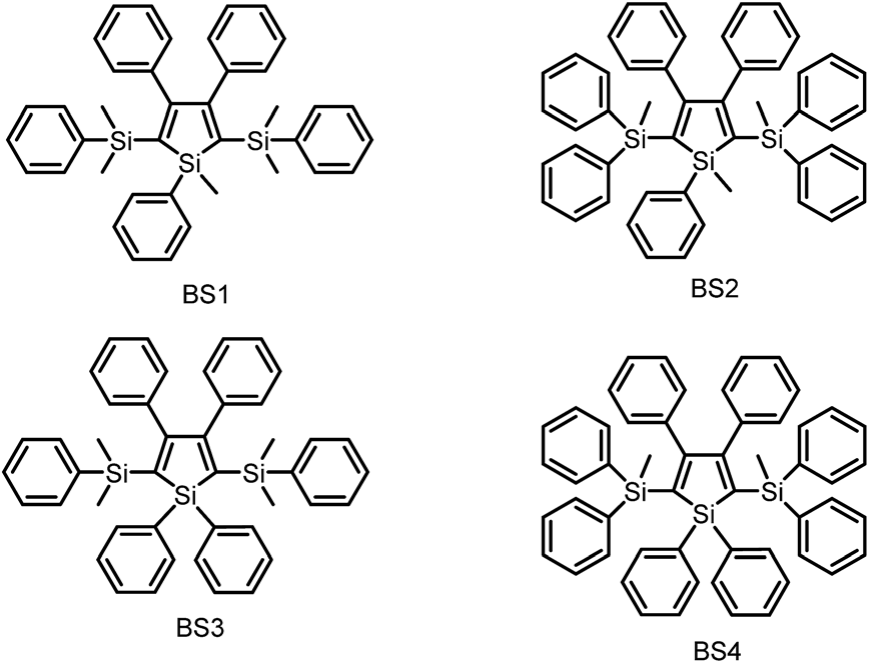
Chemical structures of siloles BS1–4.

**Fig. 2:**
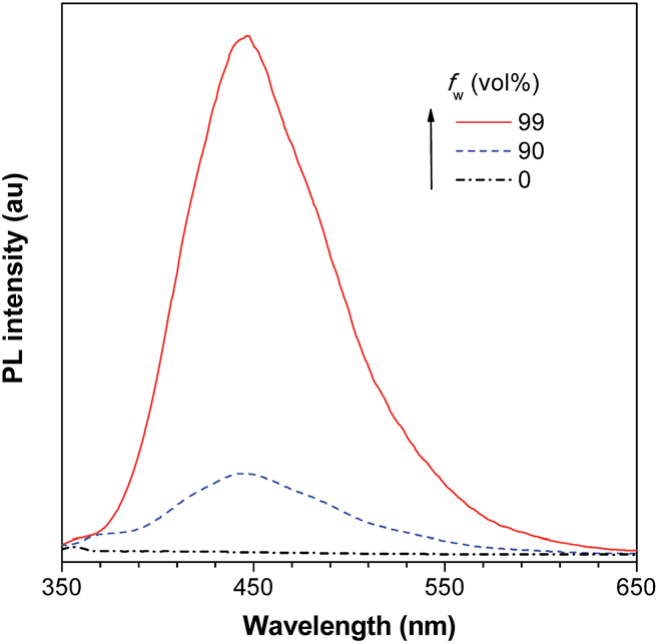
PL spectra of BS4 in THF/water mixtures with different water fractions (*f*_w_), excited at 330 nm. Reproduced with permission from ref. 25. Copyright (2013) Elsevier.

**Fig. 3:**
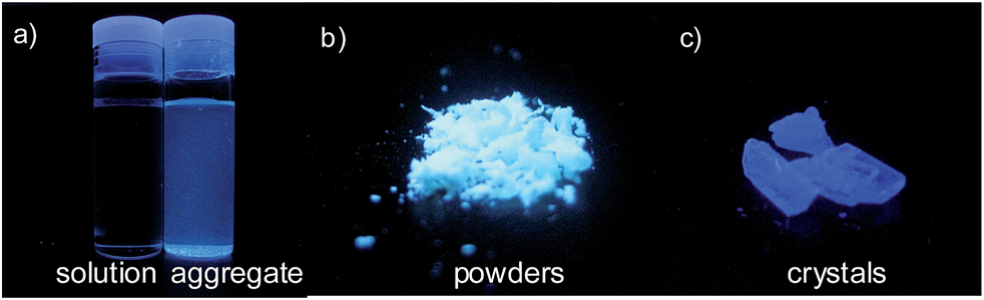
Fluorescence photographs of (a) THF and aqueous solutions, (b) powders and (c) crystals of BS4, taken under the illumination of a UV lamp (365 nm). Reproduced with permission from ref. 25. Copyright (2013) Elsevier.

As discussed above, the substituents at the 2,5-positions influence the emission wavelength greatly. However, it is still not clear whether aromatic substituents at these positions are really necessary to make a silole AIE-active. To gain deeper insight into the impact of these substituents on the emission properties of siloles, a series of flexible groups, dimethyl(phenyl)silyl and methyldiphenylsilyl, were introduced to the 2,5-positions of the silole ring (Scheme 3).^25^ The absorption maxima of these siloles are in the range of 311–320 nm, which are shorter than that of DPS, but longer than that of TPS. They are non-fluorescent in solution, but show a deep blue emission in the aggregated form or as solid films (Fig. 2 and 3). Their crystals exhibit bluish-violet light in the range of 413–421 nm, and their films show deep blue emissions peaking at 442–448 nm, which are much bluer than those of 2,3,4,5-tetraphenylsiloles. The weak interaction between the σ orbital in the silicon atom and the π orbital on the silole ring enables the silole derivatives to emit in the short-wavelength region. The *Φ*_F_ values of these siloles in THF solution were measured to be lower than 0.01%, while those of the solid films were enhanced to 12–21%, demonstrating their AIE characteristics.

Using flexible silyl groups as the substituents at the 2,5-positions can indeed prolong the conjugation of the molecule to some degree, and preserve the AIE properties of the siloles. Since the phenyl rings in the silyl groups are connected with the silole rings *via* a sp^3^ hybridized silicon bridge, the orbitals constituting the HOMOs and LUMOs of the siloles are barely distributed on these phenyl rings. The quenching effect of the rotations of these phenyl rings on the silole center is very weak. Therefore, the rotation of phenyl rings at the 3,4-positions should be mainly responsible for the faint emission in the solution state.

**Scheme 4:**
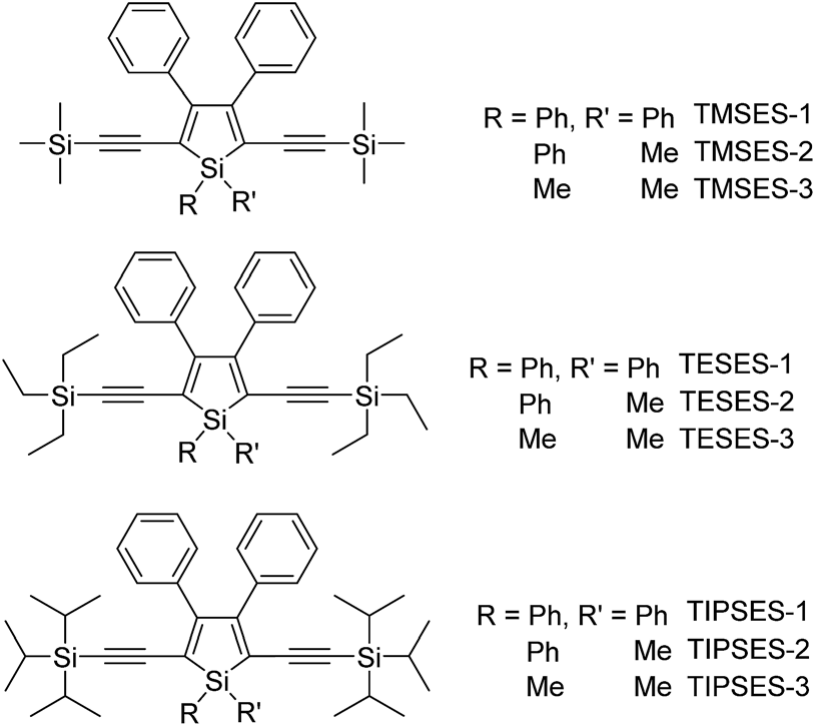
Chemical structures of siloles with (trialkylylsilyl)ethynyl groups.

To further confirm the quenching effect of the phenyl rings at the 3,4-positions, the photophysical properties of a series of siloles substituted with rod-like, π-conjugated (trialkylylsilyl)ethynyl groups, were investigated (Scheme 4).^26^ The presence of ethynyl groups effectively elongates the conjugation of the siloles, and the HOMOs and LUMOs are delocalized along the whole molecular backbone comprised of the silole ring and 2,5-ethynyl groups. The absorption maxima of these siloles are in the range of 393–403 nm, being red-shifted obviously in comparison with those of a silole with flexible silyl substituents (311–320 nm), and even those of HPS (366 nm) and MPPS (363 nm). The good conjugation across the 2,5-positions of these siloles is expected to give efficient emission. However, their emissions in solution are virtually faint, with low *Φ*_F_ values of 0.24–1.67%. The rotation of the phenyl rings at the 3,4-positions should have quenched the emission efficiently. In the solid state, their emissions are enhanced greatly, offering high *Φ*_F_ values up to 99.9%, which are much higher than those of the flexible siloles BS1–4 (12–21%), demonstrating that a rigid, well-conjugated backbone is conducive to light emission from the silole. These results also indicate that rotatable phenyl rings at the 3,4-positions of a silole ring are essential, the presence of which can keep the silole weakly fluorescent in solution, but phenyl rings at the 2,5-positions of a silole are expendable.

**Fig. 4:**
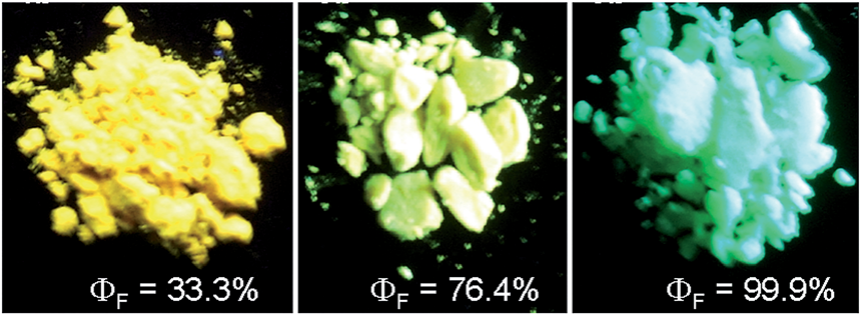
Fluorescence photos of powders of TMSES-2 (left), TESES-2 (middle) and TIPSES-2 (right), taken under the illumination of a UV lamp (365 nm). Reproduced with permission from ref. 26. Copyright (2009) Wiley-VCH.

**Fig. 5:**
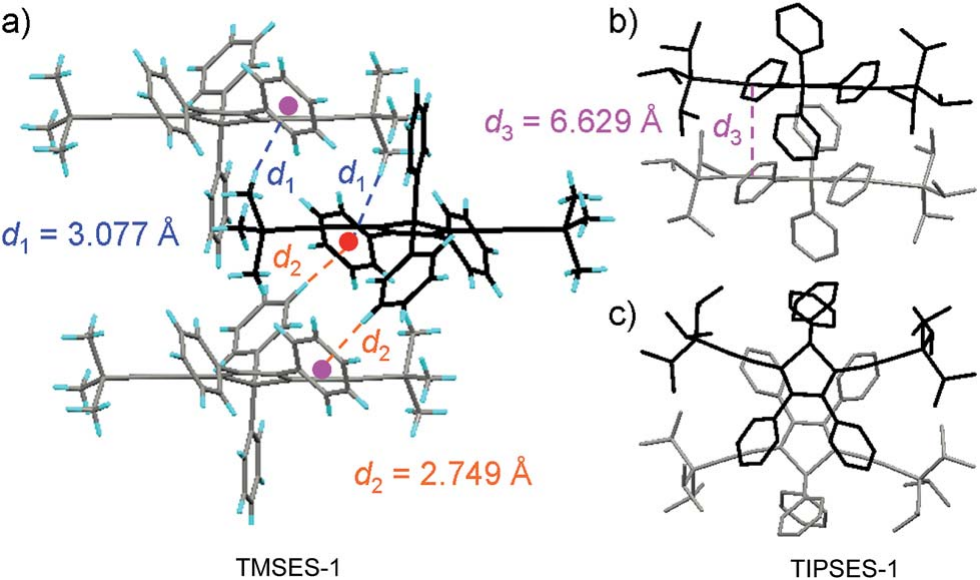
(a) Packing arrangement of TMSES-1 in the crystalline state, with C–H···π hydrogen bonds marked by dashed lines. (b) Top and (c) side views of adjacent molecules of TIPSES-1 in the crystalline state. Reproduced with permission from ref. 26. Copyright (2009) Wiley-VCH.

The peripheral alkyl silyl groups impact little on the absorption properties of siloles, and exert almost no steric hindrance of the rotation of the phenyl rings at the 3,4-positions in the solution state, due to their remote positions. Hence, these siloles show close emission wavelengths ranging from 485 to 491 nm in the solution state. However, the alkyl silyl groups can regulate well the emission wavelengths and efficiencies of siloles in their aggregated states (Fig. 4). For example, the solid of triisopropylsilyl-endcapped TIPSES-2 shows an emission peak at 489 nm and an excellent *Φ*_F_ value of 99.9%, while TMSES-2 substituted with trimethylsilyl gives a red-shifted emission at 542 nm and a decreased *Φ*_F_ value of 33.3%. Substituents with a branched structure can not only prevent the close π–π stacking of the molecules but also effectively restrict the IMR processes in the aggregated state (Fig. 5). Hence, siloles with triisopropylsilyl groups show relatively shorter emission wavelengths but much higher emission efficiencies among these siloles.

On the other hand, although substituents at the 1-position of silole rings affect little the AIE effect, they indeed have influences on the absorption maxima due to their inductive effects. The substituents at the 1,1-positions also exert apparent effects on the emission properties of siloles in the solid state. Small-sized groups allow the silole molecules to be arranged more closely, leading to redder emission colors and lower emission efficiencies.

**Scheme 5:**
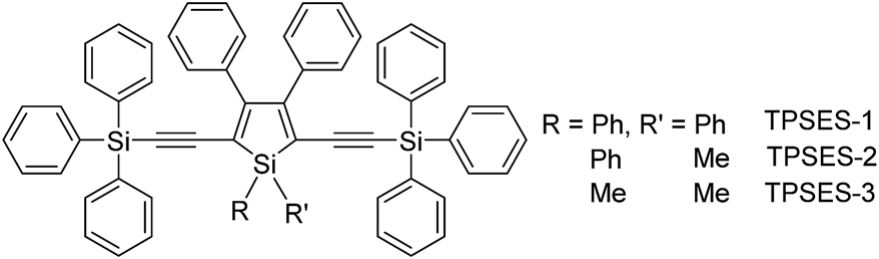
Chemical structures of siloles with (triphenylsilyl)ethynyl groups.

**Fig. 6:**
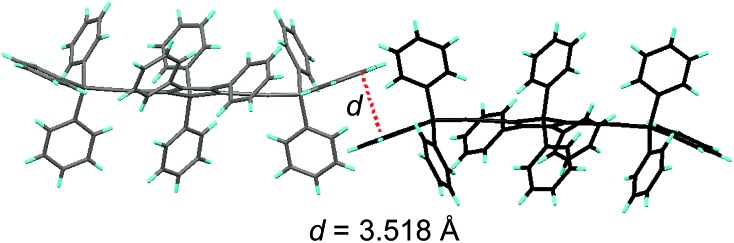
Illustration of π–π interactions between triphenylsilyl groups of adjacent molecules of TPSES-1. Reproduced with permission from ref. 27. Copyright (2011) Wiley-VCH.

Replacing the trialkylylsilyl groups with triphenylsilyl ones generates similar silole derivatives (Scheme 5) with AIE characteristics.^27^ While their emission efficiencies in the solution state are comparable to those of siloles with trialkylylsilyl groups, their solid-state emission efficiencies are lower (18.1–34.6%) than those of siloles with branched trialkylsilyl groups, such as the triisopropylsilyl ones (57.1–99.9%). This is because although the large-sized triphenylsilyl groups can prevent close stacking of the central silole ring, their phenyl rings could alternatively lead to π–π stacking in the aggregated state, which decreases the emission efficiency. The crystalline packing of these molecules has indicated the high possibility of intermolecular interactions in the solid state (Fig. 6).

### 2.2. Siloles with substituents at different positions

**Scheme 6:**
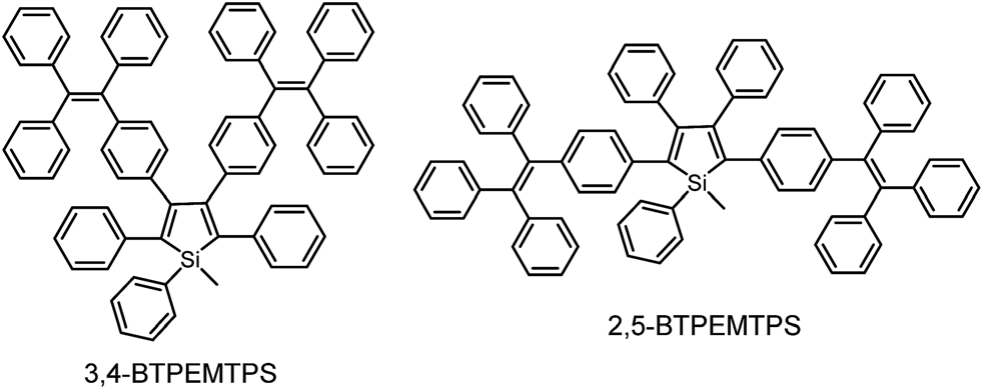
Chemical structures of 3,4-BTPEMTPS and 2,5-BTPEMTPS.

**Fig. 7:**
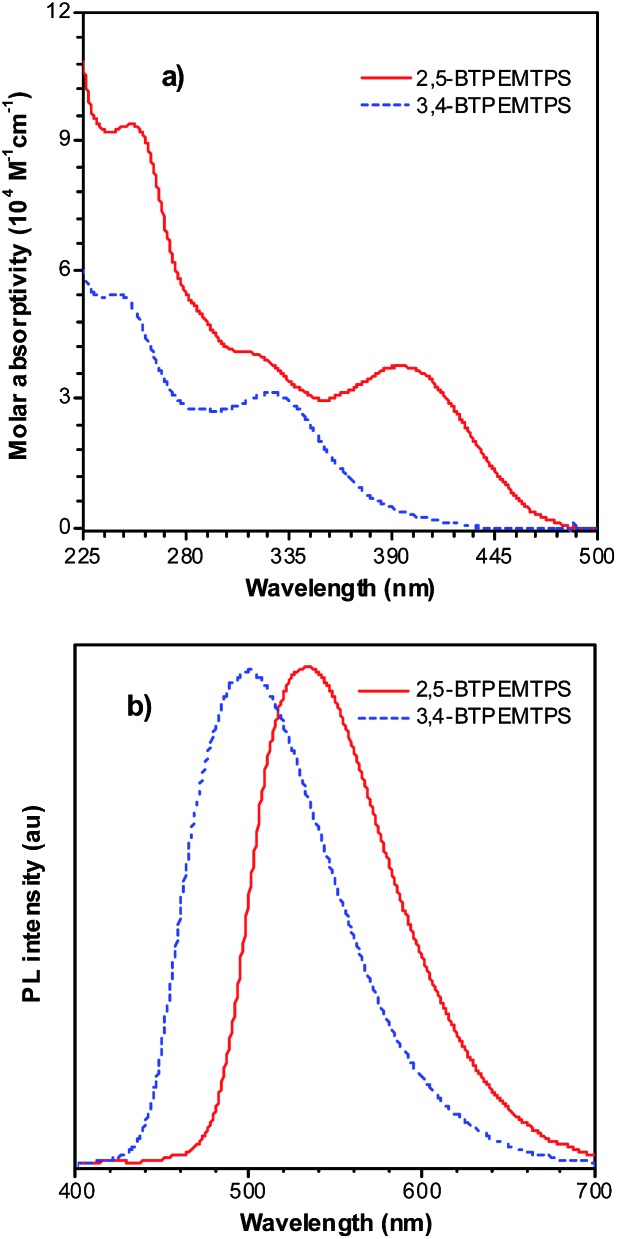
(a) Absorption spectra of 2,5- and 3,4-BTPEMTPS in THF solutions and (b) the PL spectra of amorphous films of 2,5- and 3,4-BTPEMTPS. Reproduced with permission from ref. 28. Copyright (2010) American Chemical Society.

The phenyl rotors at the 3,4-positions allow siloles to be AIE-active, while substituents at the 2,5-positions modulate the emission wavelength. So, what is the influence of prolonged conjugation at the 3,4-positions on the photophysical properties of siloles? To answer this question, 2,5- and 3,4-regioisomers integrating silole and tetraphenylethene (TPE) at the molecular level were synthesized and studied (Scheme 6).^28^ The absorption maximum of the 3,4-regioisomer, 3,4-BTPEMTPS, is at 325 nm, which is even bluer than that of its parent MPPS (363 nm). The 2,5-regioisomer, 2,5-BTPEMTPS, shows an absorption maximum at 395 nm, being 70 nm longer than that of the 3,4-regioisomer. Whereas the 3,4-regioisomer exhibits a similar emission maximum (490 nm) to that of MPPS (491 nm), the 2,5-regioisomer displays an emission maximum at much longer wavelength (520 nm) in THF solution (Fig. 7). These results clearly demonstrate that each regioisomer possesses quite a different electronic structure, and hence, exhibits varied photophysical properties. The conjugation is barely extended through the 3,4-positions and the substituents impact little on the emission wavelength of the siloles, but conjugation through the 2,5-positions is controlled obviously by the substituents, which can be utilized to tune the emission color of the siloles.

Although both silole isomers have quite different electronic structures, they are found to inherit the AIE attributes from MPPS and TPE. They show slightly higher emission efficiencies in solution than MPPS due to the enhanced steric congestion. However, the *Φ*_F_ values (0.34% and 0.38% for 2,5- and 3,4-BTPEMTPS, respectively) remain low in absolute terms, because the almost unrestricted IMR process of the multiple phenyl rotors on the periphery of the molecules can quench the light emission. As solid films, 2,5-BTPEMTPS and 3,4-BTPEMTPS show good *Φ*_F_ values of 51.2% and 46.9%, respectively. On the other hand, 2,5-BTPEMTPS shows better thermal stability than 3,4-BTPEMTPS, and also functions better in OLEDs.

### 2.3. Siloles with branched substituents

**Fig. 8:**
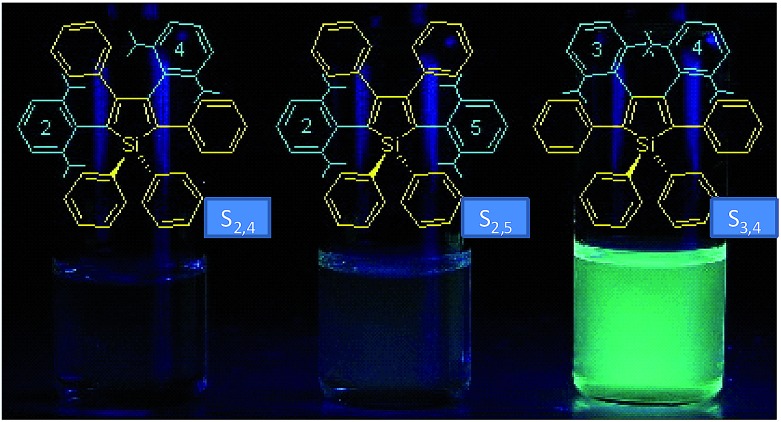
Chemical structures of isopropyl-substituted HPS derivatives, and their fluorescence photographs in THF solutions, taken under an illumination of a UV lamp (365 nm). Reproduced with permission from ref. 32. Copyright (2005) American Chemical Society.

The internal control at the molecular level of the emission behavior of luminogens is of high importance, and offers direct evidence for understanding the working mechanism behind the AIE phenomenon. Subtle modifications to the AIE molecules, such as TPE derivatives, have substantially changed the emission properties, leading to efficient light emission in the solution state.^29–31^ Li *et al.* ^32^ designed and synthesized a series of siloles with branched isopropyl substituents on the phenyl rotors (Fig. 8). The presence of the isopropyl groups has obviously changed the emission intensities of the siloles in the solution state, for these isopropyl groups give rise to increased steric congestion, which makes the rotation of the phenyl rings difficult. Therefore, the siloles with isopropyl groups show enhanced emission efficiencies relative to the siloles without isopropyl groups. However, the locations of the isopropyl groups can enhance the emission efficiencies to various extents. When the isopropyl groups are attached to the phenyl rings, the induced steric congestion results in large dihedral angles between the phenyl and silole rings, and thus, impairs the conjugation of the molecule. Therefore, silole S_2,5_ shows the bluest fluorescence in solution. The *Φ*_F_ values of the siloles with isopropyl groups located on the 2,5- or 2,4-positioned phenyl rings are 0.59 and 4.4%. For silole S_3,4_ with isopropyl groups on the 3,4-positioned phenyl rings, the *Φ*_F_ value is boosted to 69%, which is attributed to the more effective restriction of the rotation of the 3,4-positioned phenyl rings. These results further validate the rotation of the phenyl rings at the 3,4-positions of the silole ring as the dominant cause of the quenching effect.

### 2.4. Siloles with planar fluorescent substituents

According to the above discussion, the rotation of phenyl rotors, particularly those at the 3,4-positions of the silole ring, can exhaust efficiently the excited state energy, making the silole molecules weakly fluorescent in solution. However, in most cases, silole molecules are comprised of a silole ring stator and small aromatic rotors, typically phenyl rings. The emissions of these small substituents are virtually weak, and the IMR process impairs the electronic coupling between these segments. Therefore, some may doubt that the weak fluorescence of siloles in solution is caused by the damaged conjugation across the molecular backbone rather than the consumption of excited energy by the IMR process, as it is difficult to judge the quenching effect of the IMR process in these silole molecules.

**Scheme 7:**
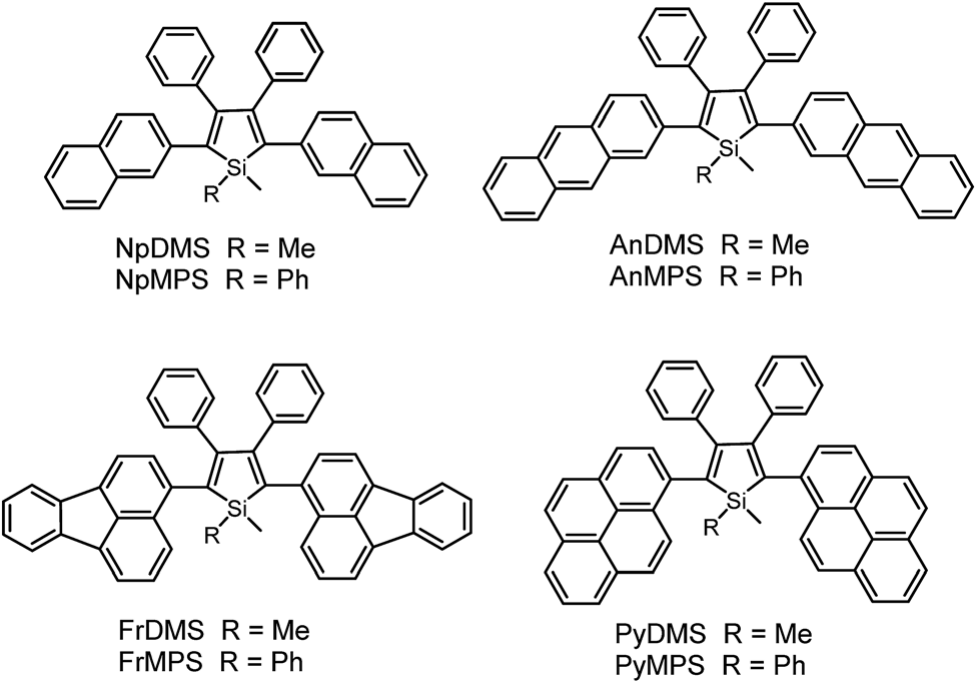
Chemical structures of silole derivatives with planar fluorescent chromophores.

Previous studies have disclosed that the IMR process of the TPE unit can effectively quench the emission of planar fluorescent chromophores (PFCs) in TPE-PFC adducts.^33^ Therefore, the emission quenching effect of the IMR process in silole systems is also envisioned. A series of PFCs that have good emission properties, such as pyrene, anthracene, naphthalene and fluoranthene, were incorporated onto the 2,5-positions of the silole ring to test the quenching effect of phenyl rotors at the 3,4-positions (Scheme 7). The results revealed that these new siloles are much weaker emitters in the solution state than PFCs existing individually, as evidenced by the much lower *Φ*_F_ values (1.2–12%) than those of the PFCs.^34^ This clearly demonstrates that the good emissions of the PFCs have been quenched by the IMR process. However, these *Φ*_F_ values are still higher than those of 2,3,4,5-tetraphenylsiloles (<0.1%), because the good emissions of PFCs compensate for the emission quenching effect of the IMR process to some degree.

**Fig. 9:**
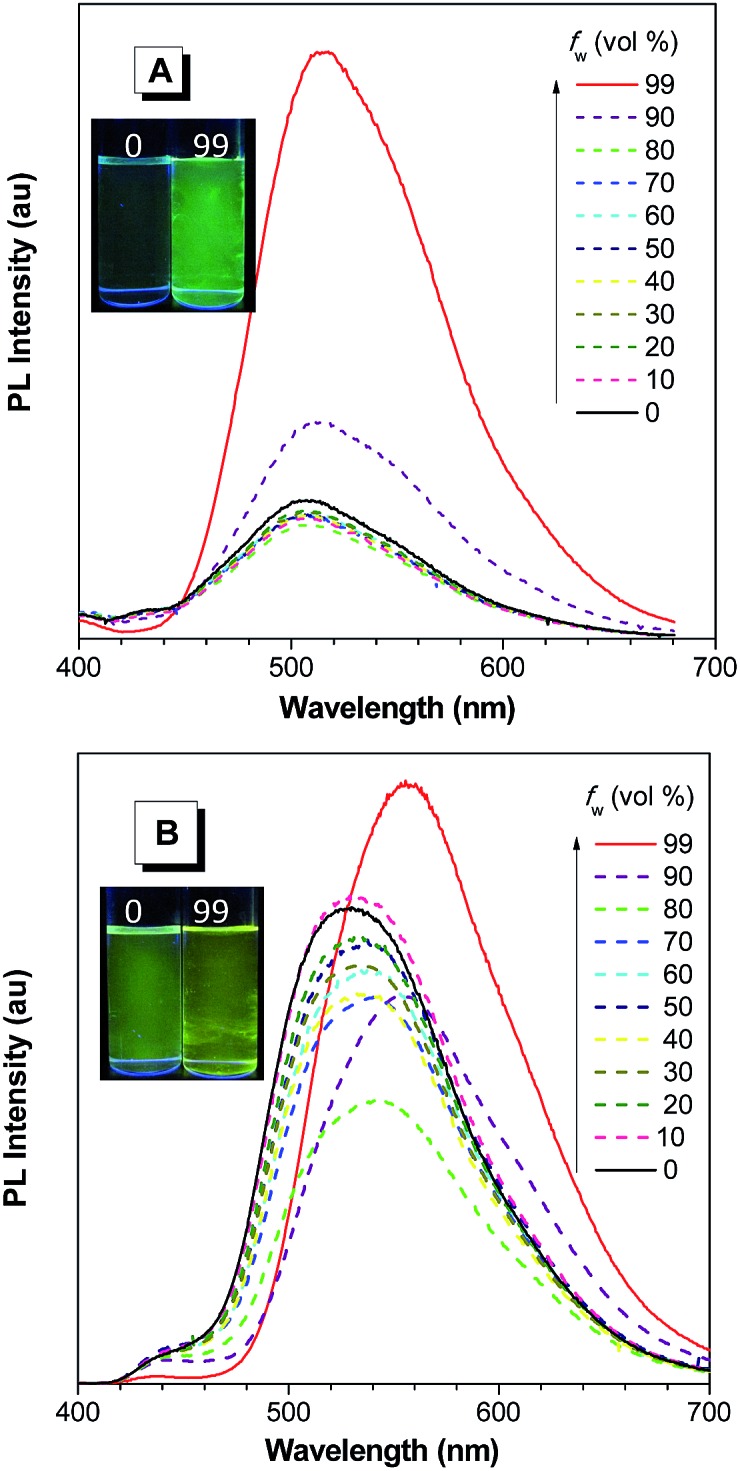
PL spectra of (A) NpMPS and (B) AnMPS in THF/water mixtures with different water fractions (*f*_w_), excited at 350 nm. Insets: photos of NpMPS and AnMPS in THF/water mixtures (*f*_w_ = 0 and 99%) taken under the illumination of a UV lamp (365 nm). Reproduced with permission from ref. 35. Copyright (2014) The Royal Society of Chemistry.

On the other hand, the conjugation of PFCs also plays an important role in determining the emission efficiency in the solution state. For example, the silole with anthracene groups (AnDMS) shows a much higher *Φ*_F_ value (12%) than that with naphthalene groups (NpDMS, *Φ*_F_ = 2.2%) in the solution state, because anthracene has more extended conjugation than naphthalene. The siloles with naphthalene groups show aggregation-enhanced emission (AEE) but those with anthracene show no apparent AIE or AEE effects (Fig. 9). Theory calculation results suggest that AnDMS and NpDMS are more rigid than DMTPS, due to better conjugation. The reorganization energies for the excited states are increased from AnDMS (2258 cm^–1^) to NpDMS (3387 cm^–1^) and to DMTPS (4920 cm^–1^),^35^ implying that the lowest nonradiative energy loss by structural relaxation from the excited state to ground state occurs in AnDMS. Thanks to the more rigid conformation, siloles with anthracene possess higher radiative decay rates but lower nonradiative decay rates than siloles with naphthalene, which leads to higher *Φ*_F_ values in solution.

If the connection between a silole ring and the PFCs becomes more twisted, due to steric congestion, the emission efficiency decreases in solution. For example, although pyrene itself is similar to anthracene in terms of effective conjugation and *Φ*_F_ value, siloles with pyrene groups show much lower *Φ*_F_ values (2.6% and 3.9% for PyDMS and PyMPS, respectively) than those with anthracene (12% and 11% for AnDMS and AnMPS, respectively). From the crystal structure of PyMPS, it can be seen that the PyMPS molecules adopt a highly twisted conformation due to steric congestion. The dihedral angles between the pyrenes and the silole ring are 89.81° and 72.36°, indicating that electronic communication between both segments becomes difficult.^37^ Concerning AnDMS and AnMPS, the anthracene and silole moieties can become relatively more coplanar, and thus, better conjugated. The different molecular conformations account for the apparent difference in the emission efficiencies of these silole derivatives.

**Fig. 10:**
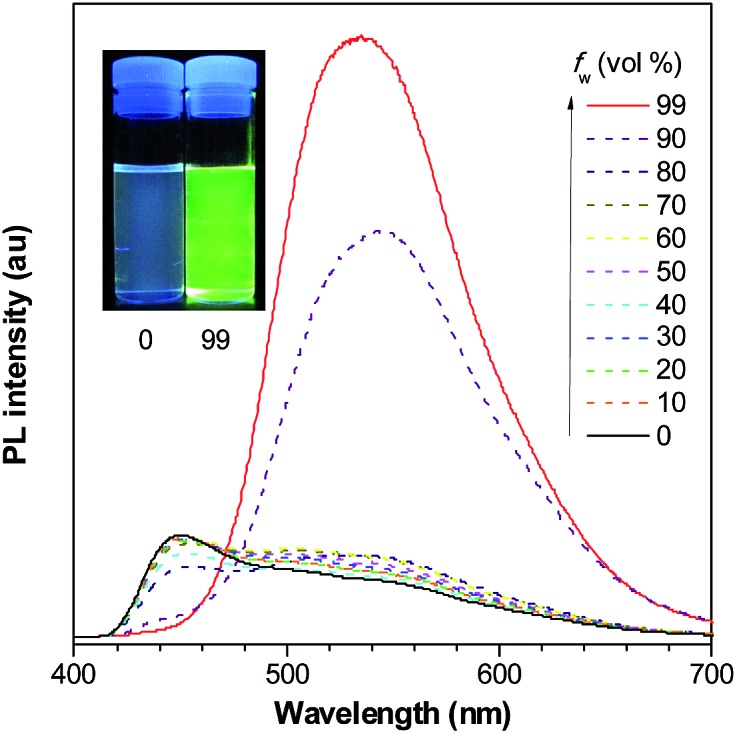
PL spectra of PyMPS in THF/water mixtures with different water fractions (*f*_w_), excited at 350 nm. Inset: photos of PyMPS in THF/water mixtures (*f*_w_ = 0 and 99%) taken under the illumination of a UV lamp (365 nm). Reproduced with permission from ref. 37. Copyright (2014) Wiley-VCH.

The introduction of large PFCs not only enhances the emission efficiencies of the siloles but also provokes obvious changes in the PL spectra in the solution state. Some siloles carrying large-sized PFCs, such as pyrene, exhibit blue emission bands (Fig. 10), which are ascribed to the emission of the pyrene moieties, in addition to the green emission band from the silole ring with certain π-extension. This can be interpreted as the IMR process leading to inefficient energy transfer from the PFC to the low-lying silole core in the solution state. The blue emission bands vanish when the molecules become more rigid in the aggregated state, where the IMR process is restricted and the electronic communication between the segments becomes dominant.^36^

**Fig. 11:**
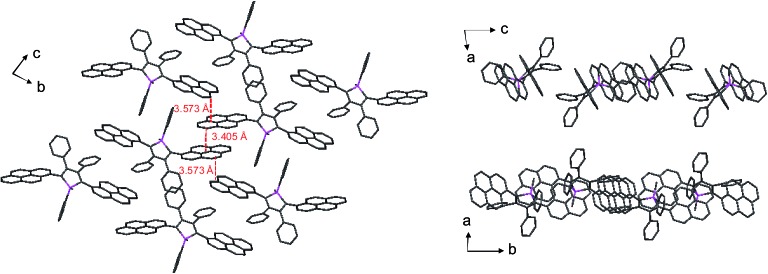
Packing pattern of PyMPS in the crystalline state. Reproduced with permission from ref. 37. Copyright (2014) Wiley-VCH.

Unlike 2,3,4,5-tetraphenylsiloles that show similar emission wavelengths in both the solution and aggregated states because of the propeller-like conformation that hampers close π–π stacking of the molecules, siloles with large PFCs give rise to red-shifted emission spectra in the aggregated state.^37^ For example, NpMPS shows an emission maximum of 515 nm as a solid film, being close to that obtained in THF solution (508 nm), with a *Φ*_F_ value of 37%. AnMPS, however, gives a much redder emission at 559 nm, with a lower *Φ*_F_ value of 14%. Since naphthalene is smaller than anthracene in volume, the propeller-like molecular conformation can effectively prevent the close packing of the naphthalene moieties in the aggregated state. Therefore, NpMPS experiences less π–π stacking interactions, and affords stable emissions. Similarly, the silole with pyrene groups (PyMPS) also gives a large red-shift as a solid film in comparison with that in the solution state. Additionally, its solid state emission efficiency (22%) is much lower than those of siloles with naphthalene and other small aromatic groups. The packing manner of PyMPS in the crystalline state manifests as the formation of multiple intermolecular π–π stacking interactions. Many sets of four pyrene moieties are piled up face to face, and the distance between the planes of two neighboring pyrene rings is 3.405 or 3.573 Å (Fig. 11). These multiple π–π stacking interactions induced by this kind of packing manner should result in spectral red-shifts and decreased emission efficiencies.

### 2.5. Siloles with different bonding patterns

**Scheme 8:**
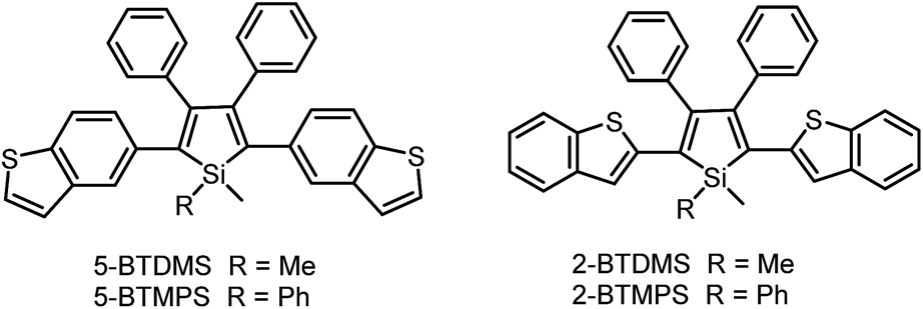
Chemical structures of siloles with benzothiophene groups.

**Fig. 12:**
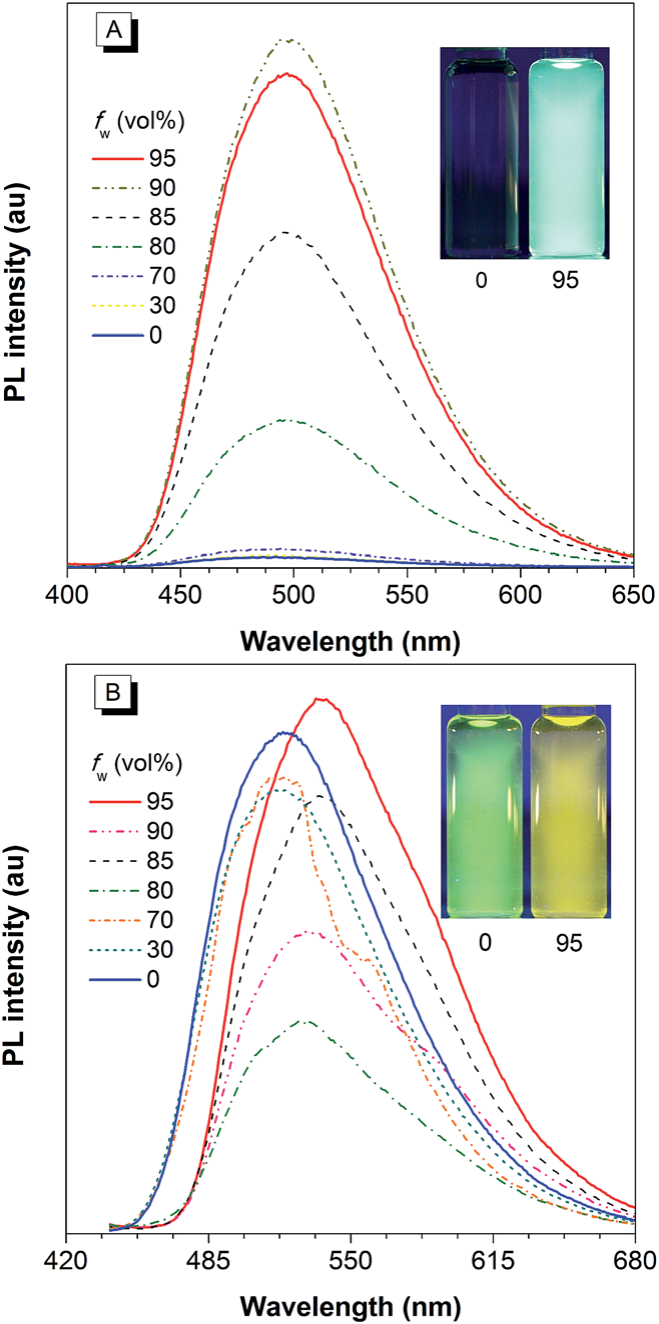
PL spectra of (A) 5-BTDMS and (B) 2-BTDMS in THF/water mixtures with different water fractions (*f*_w_). Concentration: 10 μM; excitation wavelengths: 370 nm for 5-BTDMS and 420 nm for 2-BTDMS. Insets: photos of (A) 5-BTDMS and (B) 2-BTDMS in THF/water mixtures (*f*_w_ = 0 and 95%), taken under the illumination of a UV lamp (365 nm). Reproduced with permission from ref. 38. Copyright (2015) Wiley-VCH.

The covalent bonding pattern between the substituents and a silole ring also affects the optical properties of siloles. Scheme 8 shows a series of silole derivatives containing planar benzothiophene (BT) groups at the 2,5-positions of the silole ring. Although these siloles are comprised of the same building blocks, subtle modulations to the connection patterns result in quite different conjugation degrees, which alters the optical behavior of the molecules significantly. The connection of the 2-position of the BT groups to the 2,5-positions of the silole furnishes a well-conjugated backbone within the molecules, leading to greatly red-shifted absorption maxima for 2-BTDMS (432 nm) and 2-BTMPS (437 nm) relative to 5-BTDMS (370 nm) and 5-BTMPS (375 nm).^38^ Like most 2,3,4,5-tetraphenylsilole derivatives, 5-BTDMS and 5-BTMPS show weak fluorescence peaks at 488 and 499 nm, with low *Φ*_F_ values of 0.8% and 1.2% in THF solution. Their solid films, however, are highly emissive with close emission peaks at 494 and 502 nm and high *Φ*_F_ values of 60.4% and 53.0%, revealing that they are AIE-active. 2-BTDMS and 2-BTMPS can emit more strongly in THF solution, giving emission peaks both at 522 nm and increased *Φ*_F_ values of 4.5% and 6.5%. However, as solid films, their emissions are red-shifted to 541 and 552 nm, and the *Φ*_F_ values are 5.5% and 8.5%, being barely increased relative to those in solution. Therefore, 2-BTDMS and 2-BTMPS are not typical AIE or AEE luminogens (Fig. 12).

**Fig. 13:**
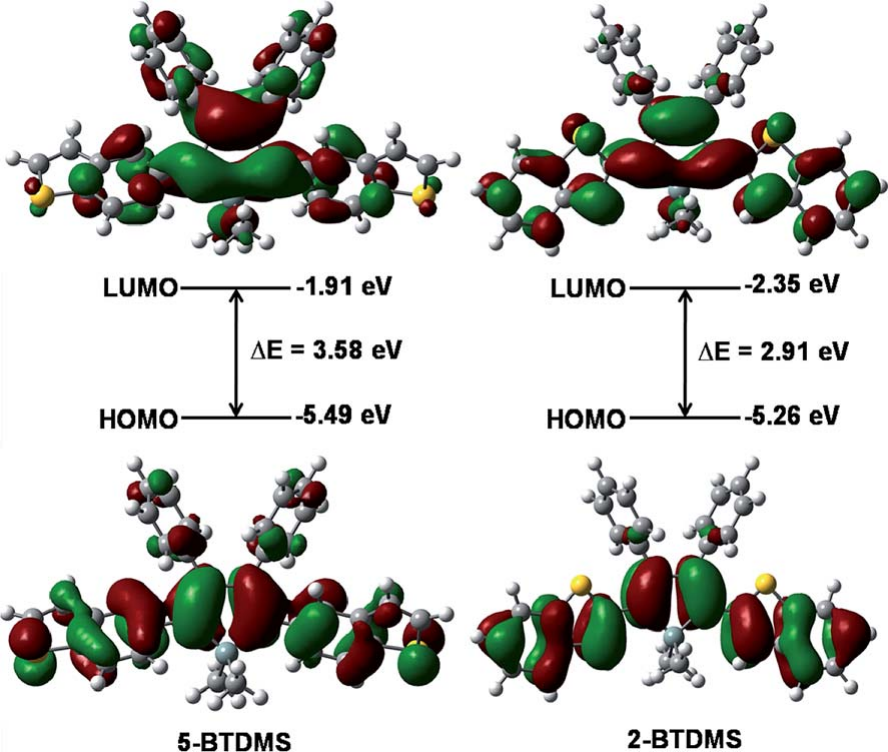
Calculated molecular orbital amplitude plots and energy levels of the HOMOs and LUMOs of 5-BTDMS and 2-BTDMS. Reproduced with permission from ref. 38. Copyright (2015) Wiley-VCH.

**Fig. 14:**
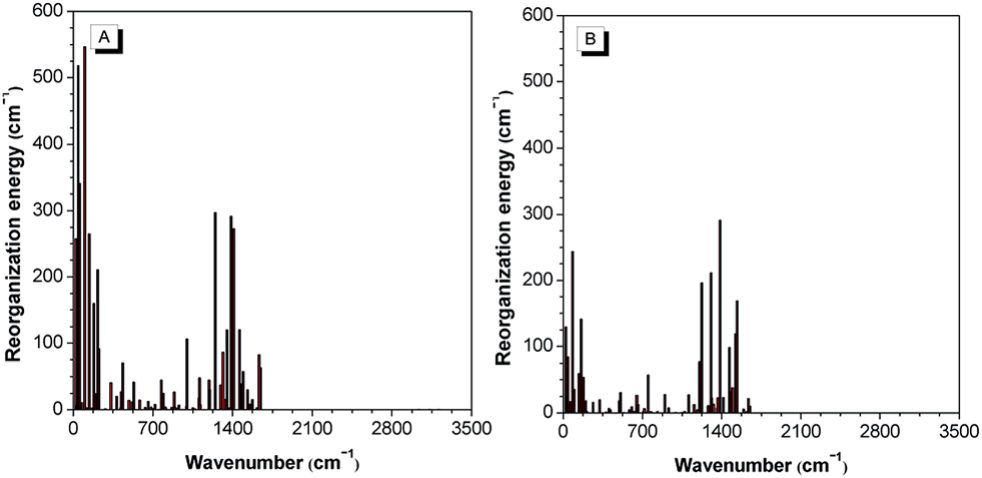
The calculated reorganization energy *versus* the normal mode wavenumber for isolated molecules of (A) 5-BTDMS and (B) 2-BTDMS. Reproduced with permission from ref. 38. Copyright (2015) Wiley-VCH.

The theoretical calculation results reveal that 2-BTDMS has a higher HOMO energy level but a lower LUMO one than 5-BTDMS (Fig. 13), corresponding to a narrower energy band gap of 2.91 eV compared with that of 5-BTDMS (3.58 eV), further validating that 2-BTDMS has better conjugation than 5-BTDMS. This is actually understandable because the thiophene ring is smaller than the phenyl ring in size. 2-BTDMS is less sterically demanding and can adopt a more coplanar conformation, which facilitates the π-electron delocalization. In the transition from the ground state (S_0_) to the first singlet excited state (S_1_), 2-BTDMS experiences a much smaller structural alteration than 5-BTDMS. 2-BTDMS also has a much smaller total reorganization energy of the S_1_ (2549 cm^–1^) than that of 5-BTDMS (4640 cm^–1^), revealing that the blocking of the nonradiative channel, the internal conversion process from S_1_ to S_0_, enhances the emission of 2-BTDMS in solution. The contribution of the low frequency modes that represent the rotational motions of the aromatic rings to the total reorganization is suppressed in 2-BTDMS in comparison with that of 5-BTDMS (Fig. 14). This also suggests that the restriction of rotational motion accounts for the good emission efficiency of 2-BTDMS in solution, and again validates the restriction of intramolecular rotation as a factor contributing to the AIE phenomenon.

The lower emission efficiencies and red-shifted emission wavelengths are caused by the well-conjugated planar backbones in 2-BTDMS and 2-BTMPS, which are prone to forming intermolecular interactions in the aggregated state. 5-BTDMS and 5-BTMPS which possess more twisted conformations can prevent strong intermolecular interactions and emit efficiently in the aggregated state. The negative effect of intermolecular interactions and the positive effect of restriction of rotational motion in the aggregated state compete against each other, and determine the emission behaviors of the molecules to a great degree.

The results presented above clearly suggest that the IMR process of the phenyl rings at the 3,4-positions can quench the light emission of luminescent chromophores attached to the silole ring. However, good conjugation between the luminescent chromophores and the silole ring, and the good emission of the substituents, can compensate for the emission quenching effect of the IMR process to some extent, and thus lead to a higher emission efficiency than siloles with small aromatic groups in the solution state. The PL emissions of siloles in the aggregated state, however, are lowered and red-shifted compared with those of 2,3,4,5-tetraphenylsiloles, due to the alternative quenching effect of the intermolecular interactions between the large PFC moieties and/or coplanar molecular backbones. Therefore, a wise choice of substituents, as well as connection position and pattern, are important in molecular design in order to achieve the desired emission color with high emission efficiency.

## 3. Potential applications

### 3.1. Chemosensors

Attracted by the intriguing AIE characteristics, more and more silole derivatives are being synthesized and their applications explored. A facile and easily handled application of siloles is as turn-on fluorescent sensors. By simple chemical modification, siloles can be utilized to detect various chemicals and metal ions. For instance, Yin *et al.* ^39^ developed a silole derivative functionalized with terpyridine anchor groups. The molecule can capture Zn^2+^ effectively, and then displays a strong red-shift in emission position and a remarkable enhancement in intensity, demonstrating a selective and sensitive turn-on sensor for Zn^2+^. Tang *et al.* ^40^ prepared a silole derivative bearing 3-formylphenyl groups at the 2,5-positions of the silole ring, and found that it was capable of discriminatively and simultaneously differentiating Cys, Hcy and GSH, relying on the differences in reaction kinetics between the aldehyde group and the analytes. Not only in their solution states, but also in their aggregated states, many silole derivatives are able to monitor chemicals. For instance, a star-shaped luminogen composed of silole peripheries and a triphenylamine core can form nanoaggregates in aqueous medium and show intensified emission.^41^ When a tiny amount of explosive, such as picric acid, is added into the aqueous solution containing the nanoaggregates, the emission is quenched efficiently and exponentially, offering a high quenching constant of 7.0 × 10^–4^ L mol^–1^.

**Fig. 15:**
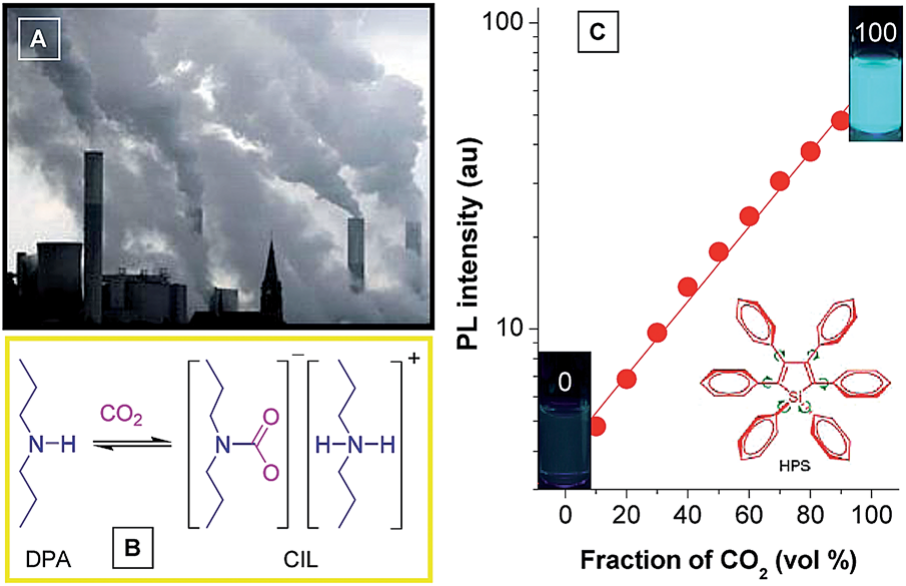
(A) Photograph showing the discharge of CO_2_ gas from a power plant into the atmosphere. (B) Scheme showing the formation of carbamate ionic liquid (CIL) by the reaction of CO_2_ with dipropylamine (DPA). (C) Plot of the emission intensity of HPS *versus* CIL fraction in the DPA/CIL mixture. Reproduced with permission from ref. 42. Copyright (2010) American Chemical Society.

Monitoring of CO_2_ gas has practical implications for environmental protection because CO_2_ gas is an important component of gas mixtures from many natural and anthropogenic processes and exerts significant influences on the global climate and human wellbeing. Although several methods have been applied for sensing CO_2_ gas, the monitoring of high-concentration CO_2_ gas basically remains untouched. Recently, Tang *et al.* developed a new approach to detect high-concentration CO_2_ gas using HPS dissolved in an amine solvent of dipropylamine (DPA) (Fig. 15).^42^ The solution is non-fluorescent without CO_2_ gas, but a strong emission is observed when CO_2_ gas is bubbled through the solution. Since CO_2_ can react with DPA to yield a carbamate ionic liquid (CIL), the viscosity of the solution is increased greatly. The IMR process in HPS is thus suppressed and radiative decay is promoted, making the solution of HPS highly emissive. As more CO_2_ gas is bubbled through the solution, more CIL is formed, which gives rise to enhancement in emission intensity. The PL intensity correlates linearly with the CO_2_ gas level (Fig. 15C), which provides an inexpensive and visually discernable method for the quantitative detection of CO_2_ gas with applications as diverse as predicting volcanic eruptions and alerting to dangerous environmental conditions.

### 3.2. Fluorescent bioprobes

**Fig. 16:**
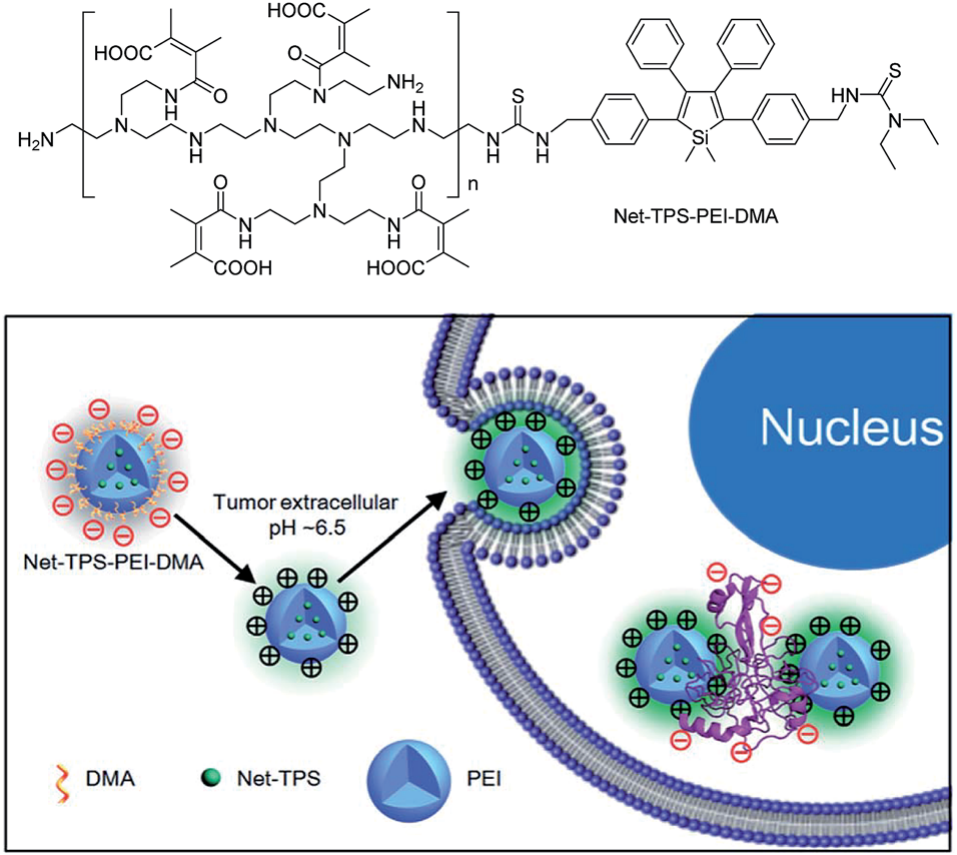
Schematic illustration of Net-TPS-PEI-DMA as a pH-responsive light-up nanoparticle probe for targeted cancer cell imaging. Reproduced with permission from ref. 43. Copyright (2014) The Royal Society of Chemistry.

Recently, silole derivatives have found highly promising applications in bioimaging, and clinical diagnostics and therapy. Liu *et al.* ^43^ smartly designed a fluorescent turn-on nanoparticle probe (Net-TPS-PEI-DMA) comprised of a 2,3,4,5-tetraphenylsilole core and a pH-responsive, charge-reversible polymeric segment. This kind of nanoparticle probe is negatively charged and almost non-fluorescent under physiological conditions (pH = 7.4). Under a tumor acidic microenvironment (pH = 6.5), the protons can bond to the amino groups in the polymeric segment and make the nanoparticles positively charged on their surfaces. Hence, due to the electrostatic interactions between the positively charged nanoparticles and negatively charged cell membranes, the nanoparticle probe is promoted to be internalized into the cancer cells. The electrostatic interactions between the positively charged residues of Net-TPS-PEI-DMA and negatively charged cancer cell membranes or cell components further accelerate the aggregation of Net-TPS-PEI-DMA, which squeezes the silole core, and thus, turns on the fluorescence. Consequently, this interesting process enables the probe to distinguish between normal and cancerous cells, and allows for specific and vivid cancer cell imaging and *in vivo* tumor tissue visualization (Fig. 16). In addition, the nanoparticles are of high cytotoxicity to cancer cells but low cytotoxicity to normal cells. Thus, they can selectively suppress and kill cancer cells in the tumor acidic extracellular microenvironment (*i.e.*, pH = 6.5), indicative of the potential as an anticancer drug.

**Fig. 17:**
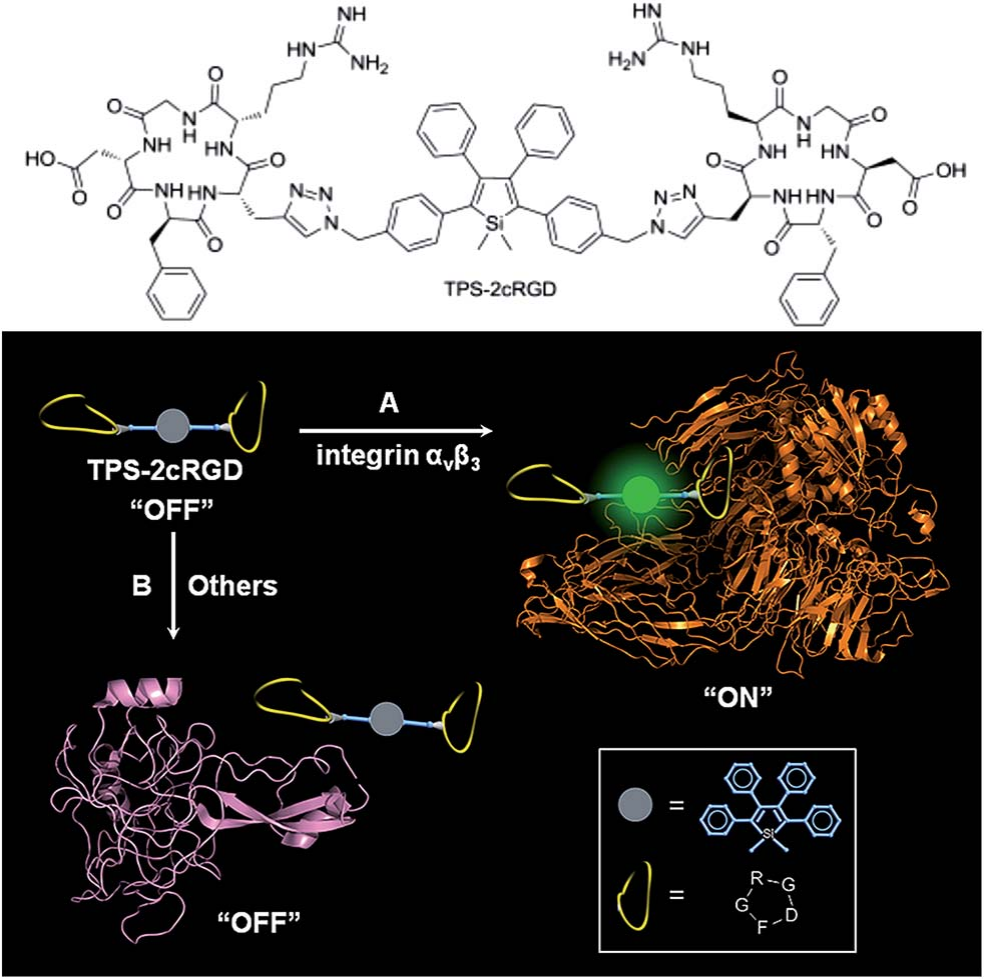
Discrimination of (A) integrin α_v_β_3_ from (B) other proteins *via* a specific cRGD-integrin interaction. Reproduced with permission from ref. 44. Copyright (2012) American Chemical Society.

Specific bioprobes with a fluorescence turn-on response were also achieved by integrating ligands such as the cyclic arginine-glycine-aspartic acid tripeptide (cRGD), which target integrin α_v_β_3_ receptors, into 2,3,4,5-tetraphenylsiloles (Fig. 17).^44,45^ Due to the AIE attributes of the 2,3,4,5-tetraphenylsilole core, the probe is almost non-fluorescent in solution. When the probes are specifically bonded to the target analyte, integrin α_v_β_3_, the IMR process in the silole core is restricted by the interactive forces between them, and the strong emission of the silole is turned on. The probe maintains an “off” state when a protein has no specific interaction with TPS-2cRGD. The probe is thus able to quantitatively detect integrin α_v_β_3_ in solution and discriminate integrin α_v_β_3_-positive cancer cells from integrin α_v_β_3_-negative ones.^45^ The binding process between cRGD and integrin α_v_β_3_ on the cells can even be imaged in a real-time manner. The specific bonding interaction between cRGD and integrin α_v_β_3_ ensures the high selectivity of the probe, which is often difficult for many probes to achieve based on a functional mechanism involving nonselective electrostatic and hydrophobic interactions between the AIE luminogens and biomolecules.

**Fig. 18:**
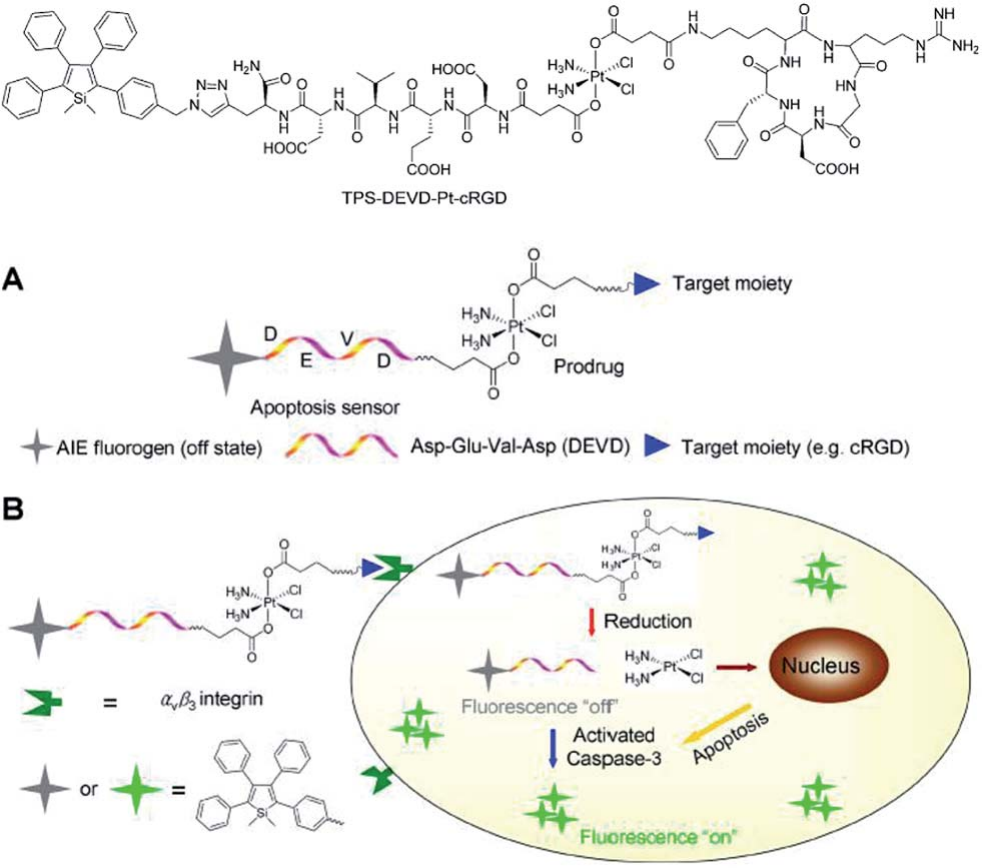
Schematic illustration of the targeted theranostic platinum(iv) prodrug with a built-in AIE-active light-up apoptosis sensor for noninvasive *in situ* early evaluation of its therapeutic response. Reproduced with permission from ref. 46. Copyright (2014) American Chemical Society.

Liu *et al.* ^46^ also successfully applied 2,3,4,5-tetraphenylsilole in the design of a chemotherapeutic Pt(iv) prodrug with a special focus on monitoring drug-induced cell apoptosis *in situ* (Fig. 18). The cyclic arginine glycine aspartic acid (cRGD) tripeptide for targeting integrin α_v_β_3_ over-expressed cancer cells and a caspase-3 enzyme specific Asp-Glu-Val-Asp (DEVD) peptide were combined to construct the prodrug. The Pt(iv) prodrug can be reduced to active Pt(ii) inside the cells and simultaneously releases the cell apoptosis sensor composed of 2,3,4,5-tetraphenylsilole and DEVD. The generated Pt(ii) causes apoptosis of the cancer cells and activates caspase-3, which further cleaves the DEVD sequence within the apoptosis sensor. Therefore, the hydrophobic interaction between the 2,3,4,5-tetraphenylsilole segments leads to aggregate formation, which turns on light emission.^46^ This prodrug system with AIE characteristics allows the early evaluation of its therapeutic response in cancer cells with high sensitivity.

### 3.3. Two-photon fluorescence bioimaging

Two-photon fluorescence (TPF) microscopy is a highly promising noninvasive technology for living cell and tissue imaging, due to a number of merits including increased penetration depth, higher spatiotemporal resolution, diminished tissue autofluorescence interference, and reduced photodamage as compared to one-photon fluorescence imaging. Robust luminescent reagents with strong two-photon absorptions in the near-infrared (NIR) region (700–1000 nm) are highly desirable for TPF biological imaging applications. In addition, high *Φ*_F_ values of the luminescent reagents are also preferable for high-contrast, vivid TPF images, but those suitable for organic dot fabrication are still rare because the emissions of many conventional chromophores are lowered greatly in the dot state because of the ACQ effect. In view of this, AIE-active luminogens are ideal candidates for the fabrication of organic dots. Although AIE-active molecules are weakly fluorescent when molecularly dispersed in solution, their aggregates can fluoresce strongly, allowing for high *Φ*_F_ values. Meanwhile, the loaded concentration of AIE-active molecules can be maximized to reach a large two-photon absorption cross section value per aggregated unit.

**Fig. 19:**
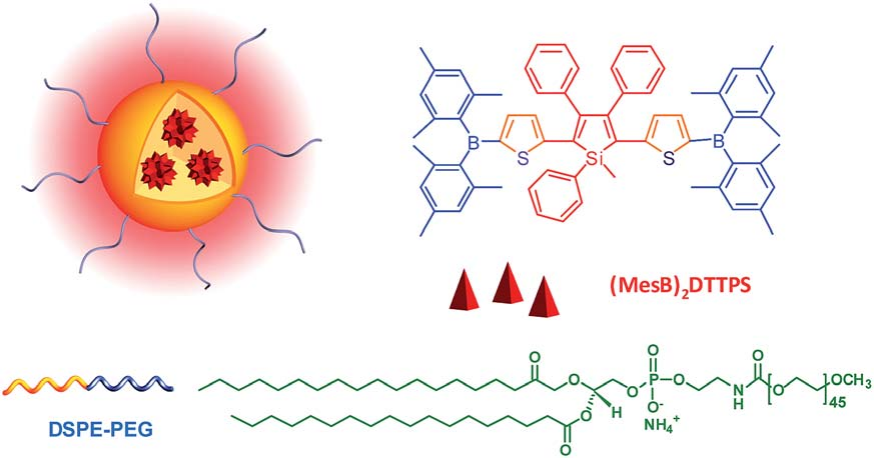
Chemical structure of (MesB)_2_DTTPS and schematic illustration of organic dots based on (MesB)_2_DTTPS.

**Fig. 20:**
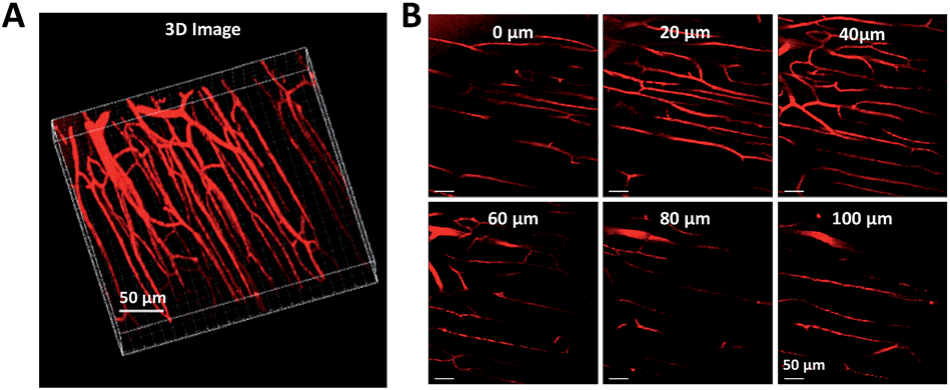
Two-photon fluorescence images of (MesB)_2_DTTPS dot-stained muscle blood vessels. (A) 3D reconstructed image of blood vessels in muscle. (B) Images at different vertical depths of mouse muscle.

Previous studies have shown that the nanoparticles of AIE-active siloles can stain cells for one-photon fluorescence cellular imaging and long term tracking.^47^ However, robust siloles with potential utility in TPF imaging applications are seldomly reported, possibly due to the green fluorescence of most siloles, which is not preferable for TPF imaging. Recently, a red fluorescent silole, (MesB)_2_DTTPS, carrying (dimesitylboranyl)thiophen-2-yl substituents was prepared (Fig. 19).^48^ The intramolecular charge transfer process is strengthened because of the good electron-donating ability of the thiophene units, which gives rise to the red fluorescence of the molecule. (MesB)_2_DTTPS shows weak emission peaking at 568 nm with a *Φ*_F_ value of 5.6% in THF solution. The organic dots fabricated by encapsulating (MesB)_2_DTTPS within DSPE–PEG matrix emit a red fluorescence located at 598 nm and give a high *Φ*_F_ value of 32%, indicative of the AEE attribute. Meanwhile, a large two-photon absorption cross section of 3.43 × 10^5^ GM, and a two-photon action cross section of 1.09 × 10^5^ GM, at an excitation wavelength of 820 nm, are recorded per dot, revealing their great potential as luminescent reagents for TPF imaging. The (MesB)_2_DTTPS dots are biocompatible, and can be applied in the one-photon and two-photon fluorescence targeted imaging of MCF-7 breast cancer cells after functionalization of the dot surfaces with a cell penetration peptide. The dots also function well in the TPF *in vivo* real-time visualization of blood vessels in mouse muscle (Fig. 20) and ear skin in a vivid and noninvasive manner, providing abundant spatiotemporal information about the three-dimensional network of blood vascular structures.

### 3.4. Self-assembled chiral materials

Chiral materials with efficient circularly polarized luminescence (CPL) are of particular interest, due to their important applications in biological detectors and optoelectronic devices. The emission dissymmetry factor *g*_em_ and fluorescence efficiency *Φ*_F_ are two essential parameters for judging the nature of a chiral luminescent material. Most CPL-active materials in the literature only show good properties in solution, but inferior performance when existing in the aggregated phase. The CPL signals are weakened because the emission of a chiral chromophore is normally quenched by aggregate formation. Hence, it remains a challenging task to develop solid-state luminescent materials with strong CPL signals.

**Scheme 9:**
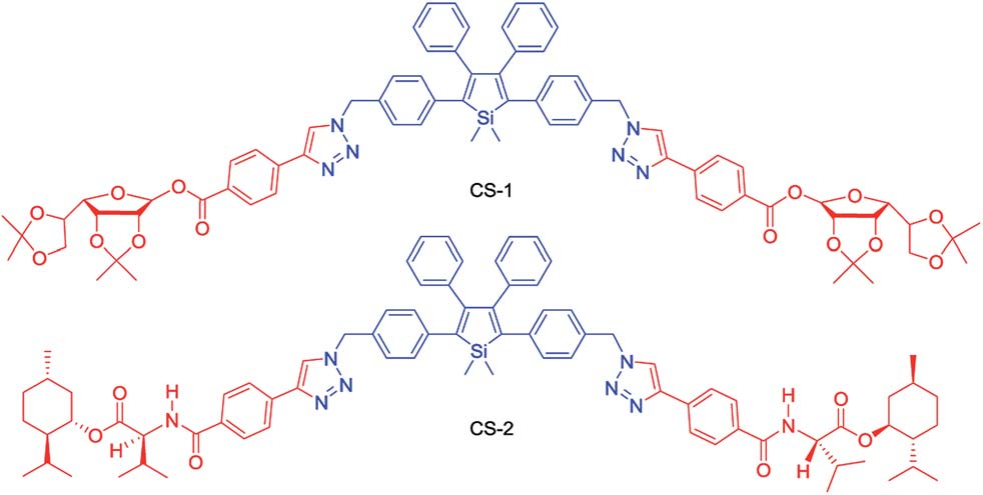
Chemical structures of siloles carrying chiral groups.

**Fig. 21:**
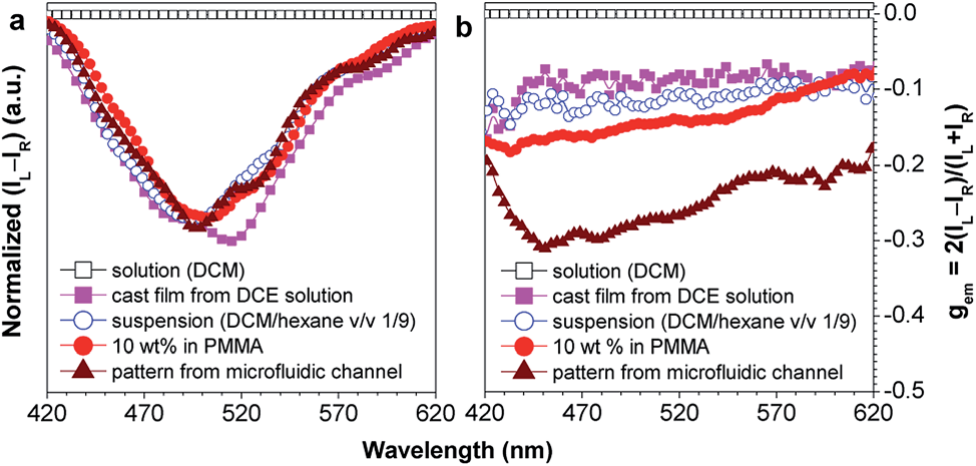
Plots of (a) (*I*_L_ – *I*_R_) and (b) the CPL dissymmetry factor *g*_em_ *versus* wavelength for CS-1 existing in different formats: DCM solution, DCM/hexane (v/v 1/9) mixture (suspension), neat cast film from a DCE solution of 2 mg mL^–1^, dispersion in a polymer matrix (10 wt% in PMMA), and a fabricated micropattern by evaporation of DCE solution in microfluidic channels; *g*_em_ = 2(*I*_L_ – *I*_R_)/(*I*_L_ + *I*_R_), where *I*_L_ and *I*_R_ denote the left- and right-handed emission intensities, respectively. In DCM and the DCM/hexane mixture, the concentration was 2 × 10^–4^ M and the excitation wavelength was 325 nm. Reproduced with permission from ref. 49*a*. Copyright (2012) The Royal Society of Chemistry.

**Fig. 22:**
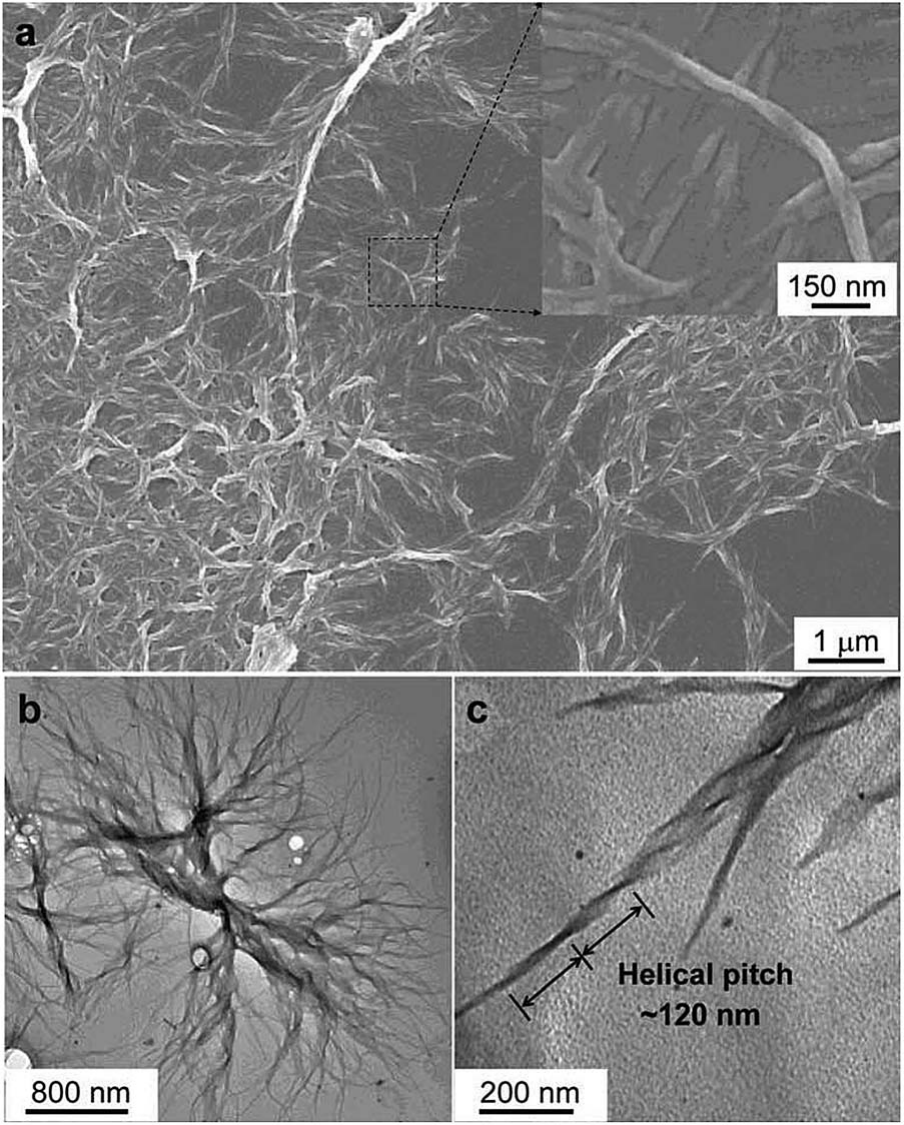
Characterisation of the microstructures of the assemblies of CS-1. (a) SEM images of the aggregates of CS-1 in the DCM/hexane mixture (v/v 1/9). Concentration: 2 × 10^–4^ M. Inset: enlarged area as indicated. (b and c) TEM images of the aggregates of CS-1 in the DCM/hexane mixture (v/v 1/9). Reproduced with permission from ref. 49*a*. Copyright (2012) The Royal Society of Chemistry.

Recently, Tang *et al.* prepared two silole derivatives, CS-1 and CS-2, consisting of an AIE-active 2,3,4,5-tetraphenylsilole core and chiral pendants (sugar and valine) connected to each other by a triazole group (Scheme 9).^49^ Both luminogens turned out to be high-performance CPL-active materials with large *Φ*_F_ and *g*_em_ values in their aggregated states. They show neither circular dichroism (CD) signals nor fluorescence in the solution state. However, as the aggregate forms, obvious CD signals and intense fluorescence are recorded. CS-1 tends to emit right-handed circular polarized (RCP) light when aggregated as nanoparticles in suspension, fabricated into thin films, or dispersed as domains in a polymeric matrix, as well as when assembled into a regular micropattern (Fig. 21), with large *g*_em_ values of up to –0.32. In addition, these molecules can undergo a self-assembly process to form varied nanostructures. CS-1 can self-assemble into right-handed helical nanostructures, such as nanoribbons and superhelical ropes (Fig. 22). The interesting CD signals and circular polarized emissions are considered to be caused by the chirality transfer from the chiral groups to the silole core, which forces the silole core to be helically arranged in a preferred screw manner. In the solution state, the silole molecules are conformationally random due to the IMR process, which suppresses the CPL signals.

**Fig. 23:**
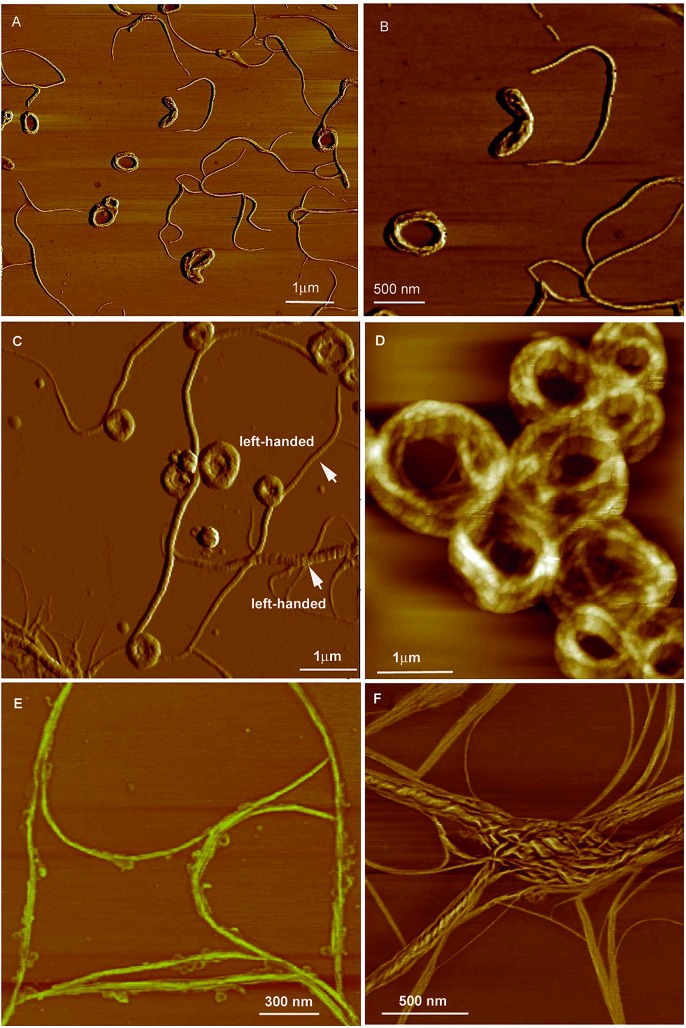
AFM images of the helical assemblies formed by CS-2 upon the evaporation of its different THF/water mixtures. The water content is (A and B) 5%, (C and D) 20%, (E) 80%, and (F) 90%. Reproduced with permission from ref. 49*b*. Copyright (2014) The Royal Society of Chemistry.

CS-2, which bears valine groups, also shows aggregation-induced CD and PL. Left-handed circular polarized (LCP) light was detected from the casted film, which was not detected in the solution state. The average *g*_em_ value is about –0.5 in the spectral window range of 420–620 nm. The presence of chiral amino acid groups also exerts an asymmetric force field on the silole core and induces a helical molecular conformation when the IMR process is suppressed in the condensed phase. CS-2 is assembled into left-handed nanostructures by the slow evaporation of THF solvent. Addition of a poor solvent, water, to the THF solution of CS-2 can also cause left-handed nanostructures, which vary from thin nanofibers to thicker loops as the fraction of water increases (Fig. 23). But the addition of hexane to the THF solution can give rise to handedness inversion of the nanomaterials, and afford right-handed nanofibers, suggesting that the polarity of the mixtures impacts the self-assembling behaviors of the molecules.

### 3.5. Organic light-emitting diodes

2,3,4,5-Tetraphenylsiloles are excellent solid-state luminescent materials due to their intriguing AIE characteristics, and thus, are expected to be outstanding light-emitting materials for OLEDs. Since the pioneering work done by Tang *et al.* on the fabrication of OLEDs with MPPS,^19a,50^ a great many AIE-active silole-based luminescent materials have been developed and utilized in OLEDs. However, most OLEDs based thereon afford moderate external quantum efficiencies around 3%. Only limited silole-based OLEDs were reported to have high electroluminescence (EL) performances with efficiencies approaching the theoretical limits.^51^

**Scheme 10:**
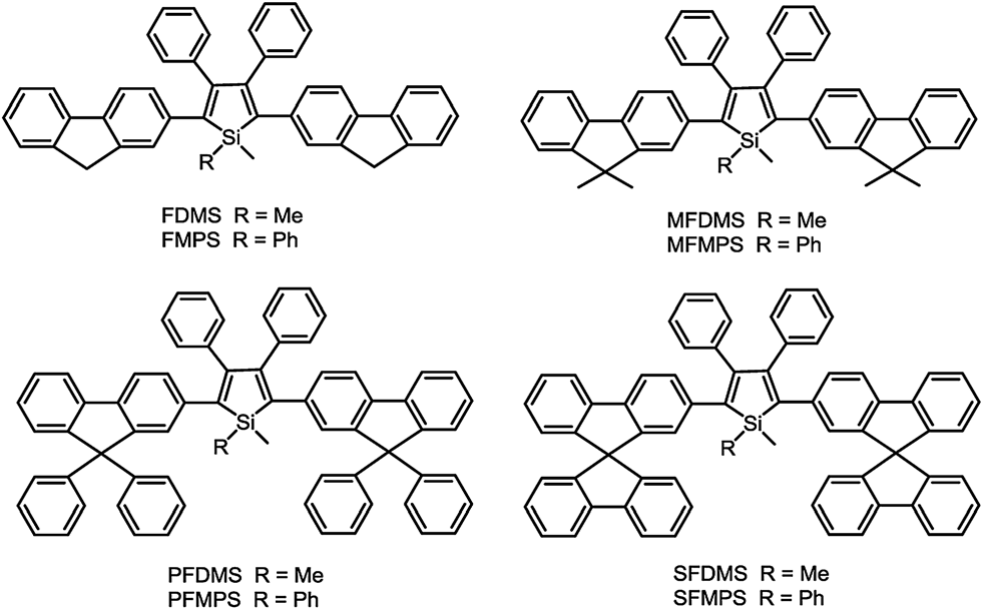
Chemical structures of siloles substituted with different fluorene derivatives.

Recently, with the great endeavors of molecular engineering, efficient light-emitting materials for OLEDs have been created from silole derivatives. By the incorporation of fluorene-based substituents, including bare fluorene, 9,9-dimethylfluorene, 9,9-diphenylfluorene and 9,9′-spirobifluorene, a series of efficient solid-state light emitters have been generated (Scheme 10).^52^ These silole derivatives show absorption maxima in the range of 388–402 nm, and main emission peaks in the range of 497–515 nm, accompanied by blue emission bands (409–439 nm) stemming from the fluorene groups. The *Φ*_F_ values in solution are slightly enhanced from 2.5 to 5.4% as the volume of the substituents increases. The films of these silole derivatives fluoresce solely at 521–535 nm, and the blue emission peaks disappear, due to the restriction of the IMR process and rigidified molecular backbones. The solid films are also highly emissive, giving high *Φ*_F_ values of 50–88%.

**Fig. 24:**
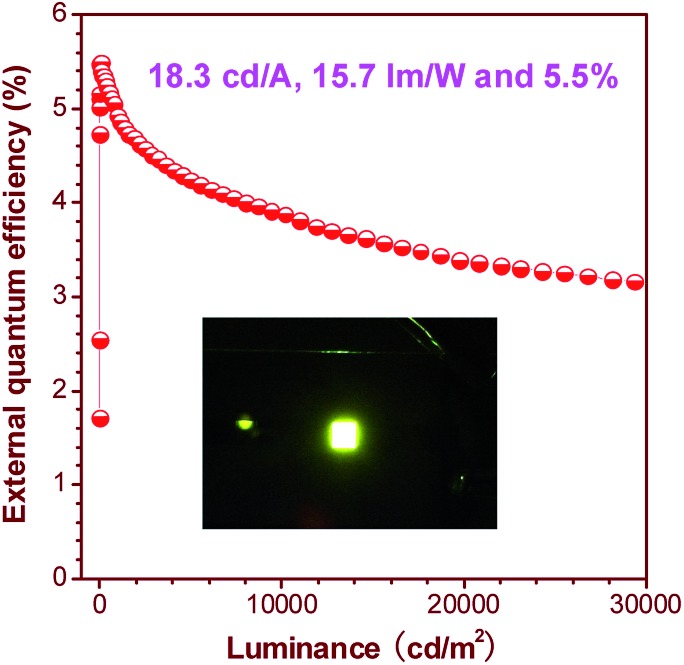
Plot of the external quantum efficiency *vs.* luminance of an OLED based on MFMPS. Inset: photo of the OLED. Reproduced with permission from ref. 52. Copyright (2014) Wiley-VCH.

The OLEDs based on these silole derivatives exhibit high performances. The undoped OLED of MFMPS (ITO/NPB (60 nm)/emitter (20 nm)/TPBi (40 nm)/LiF (1 nm)/Al (100 nm)) radiates yellow light at 544 nm (CIE = 0.37, 0.57), and affords the best EL performance with a lowest turn-on voltage (*V*_on_) of 3.2 V, and a highest maximum luminance (*L*_max_) of 31 900 cd m^–2^. The maximum current efficiency (*η*_C,max_), maximum power efficiency (*η*_P,max_) and maximum external quantum efficiency (*η*_ext,max_) attained by the device are as high as 16.0 cd A^–1^, 13.5 lm W^–1^ and 4.8%, respectively. By increasing the thickness of the TPBi layer to balance the holes and electrons and adding a thin layer of MoO_3_ to facilitate the hole-injection, an even more efficient OLED (ITO/MoO_3_ (5 nm)/NPB (60 nm)/MFMPS (20 nm)/TPBi (60 nm)/LiF (1 nm)/Al (100 nm)) was obtained, which shows a *V*_on_ of 3.3 V, a yellow light peak at 540 nm (CIE = 0.36, 0.57), and excellent luminance, current and power efficiencies of 37 800 cd m^–2^, 18.3 cd A^–1^ and 15.7 lm W^–1^, respectively. Impressively, a notable *η*_ext,max_ of 5.5% was achieved by the device (Fig. 24), which exceeds the theoretical limit for EL devices based on conventional fluorescent materials.

Thanks to the unique electronic structure of σ*–π* conjugation, siloles possess lower-lying LUMO energy levels, and higher electron affinities than common five-membered heterocycles, such as pyrrole, furan and thiophene, and are considered as a new class of electron-transporting materials. However, as a matter of fact, bare 2,3,4,5-tetraphenylsiloles are still not capable of functioning as electron transporters in OLEDs. The incorporation of other electron-withdrawing groups, such as pyridine, can improve the electron-transporting ability and enable them to function as electron-transporting layers.^18*a*^ However, those that can simultaneously serve as light-emitting and electron-transporting layers are rarely developed. West *et al.* ^53^ introduced diquinaldinatoalumino groups onto the 1,1-positions of 2,3,4,5-tetraphenylsiloles to improve their electron-transporting abilities. However, the EL performances of the non-doped devices prepared from the resulting siloles were hardly advanced.

**Scheme 11:**
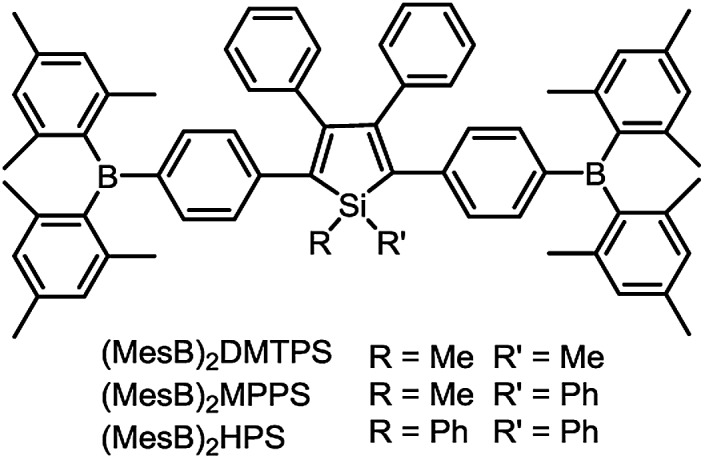
Chemical structures of siloles carrying bimesitylboryl groups.

**Fig. 25:**
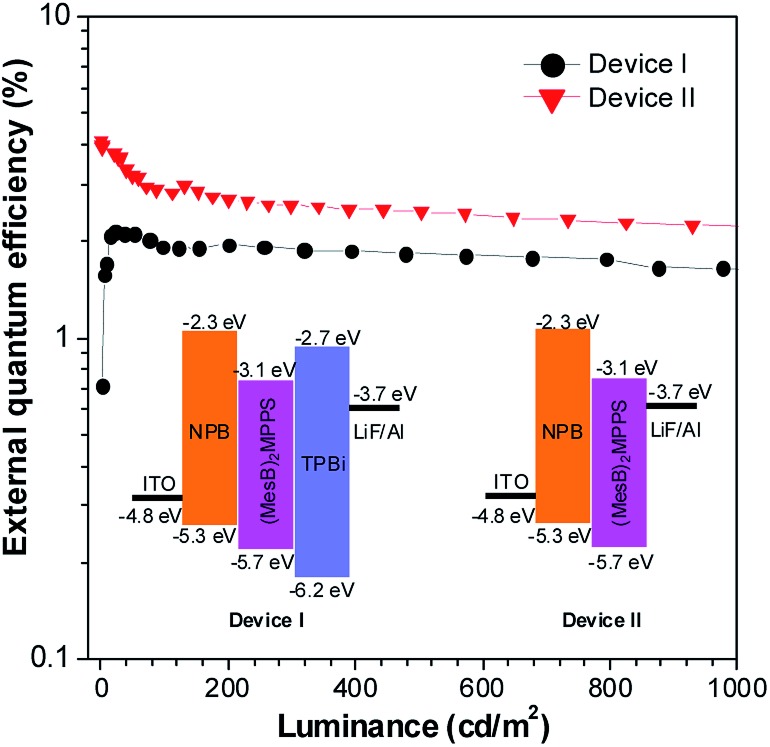
Plots of the external quantum efficiency *vs.* luminance of devices based on (MesB)_2_MPPS. Inset: device configuration and energy levels. Reproduced with permission from ref. 54. Copyright (2014) Wiley-VCH.

**Scheme 12:**
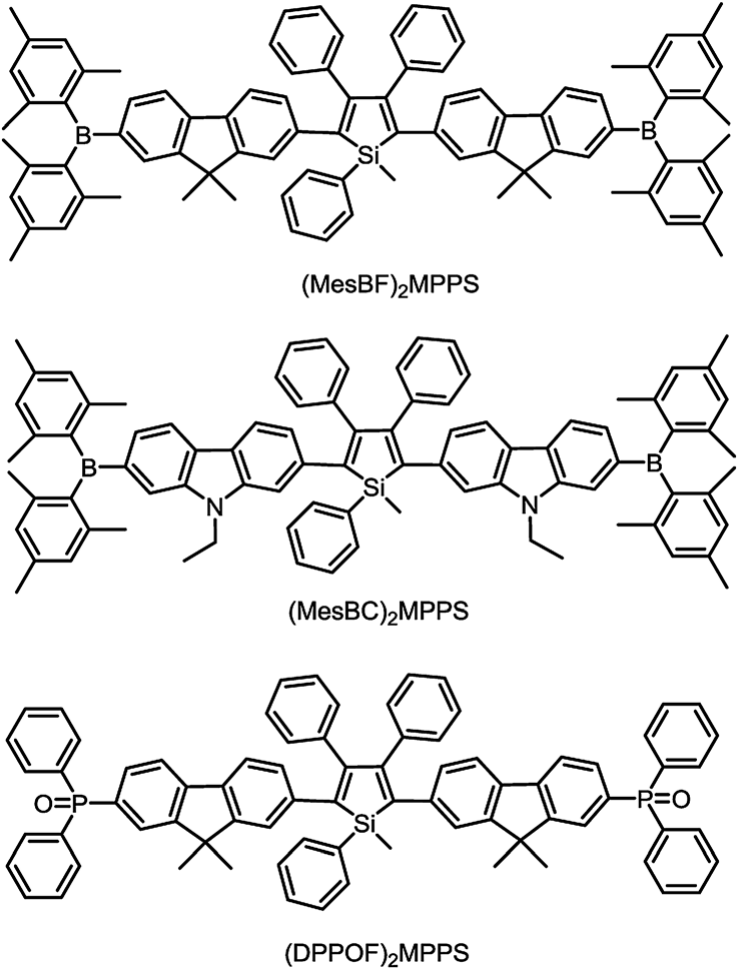
Chemical structures of siloles carrying bimesitylboryl or diphenylphosphine oxide groups.

To address this issue, a series of tailored dimesitylboryl functionalized silole derivatives were developed (Scheme 11).^54^ The grafting of inherently electron deficient bimesitylboryl groups is beneficial in advancing the electron affinity and electron-transporting ability of the molecule. In addition, the branched conformation of the bimesitylboryl group can suppress close π–π stacking, and thus keep the AIE characteristics intact. These silole derivatives are highly emissive as solid films, with emission peaks at 516–526 nm and high *Φ*_F_ values of 56–62%. The LUMO energy levels of (MesB)_2_DMTPS, (MesB)_2_MPPS and (MesB)_2_HPS are –3.00, –3.06 and –3.10 eV, respectively, which are equal to or even lower than those of commercialized typical electron transporters, such as tris(8-hydroxyquinoline)aluminum (Alq_3_, –3.0 eV) and 1,3,5-tris(*N*-phenylbenzimidazol-2-yl)benzene (TPBi, –2.7 eV), revealing that they are capable electron-transporting materials for OLEDs (Fig. 25). A similar silole derivative, (MesBF)_2_MPPS, created by integrating dimesitylboryl substituents into MFMPS, also shows excellent solid-state emission and great potential as an electron transporter.^55^ The incorporation of other electron deficient substituents, such as diphenylphosphine oxide, can also improve the electron-transporting ability without undermining the solid-state fluorescence efficiency (Scheme 12).

The double-layer OLEDs [ITO/NPB (60 nm)/silole (60 nm)/LiF (1 nm)/Al (100 nm)] fabricated by adopting (MesB)_2_MPPS or (MesB)_2_HPS as both light emitter and electron transporter show EL emissions at 520 nm (CIE = 0.30, 0.56) and 524 nm (CIE = 0.33, 0.56), and afford excellent performances, with high EL efficiencies up to 13.9 cd A^–1^, 4.35% and 11.6 lm W^–1^. The values are much higher than those attained from the triple-layer devices with an additional TPBi electron-transporting layer (8.4 cd A^–1^, 2.62% and 4.1 lm W^–1^). The advanced EL performances of both silole derivatives are attributed to the improved electron-transporting ability of the materials, which balances holes and electrons in the devices, and thus enhances the carrier recombination efficiencies. Thanks to the matched energy levels and electron-transporting abilities of the present siloles, the TPBi layer is actually not necessary, and may even have an adverse effect on device performance because of the contact resistance at the interfaces. These results demonstrate that these dimesitylboryl functionalized siloles are promising bifunctional materials, n-type solid-state light emitters, for the fabrication of high-performance OLEDs with simplified device structures.

By a similar method, p-type solid-state luminescent materials that function remarkably in double-layer OLEDs were also achieved.^56^ The silole carrying the triphenylamine groups, 1,1-dimethyl-2,5-bis(4-diphenylaminophenyl)-3,4-diphenylsilole (DMTPS-DPA), shows not only a high solid-state fluorescence efficiency (74%) but also a good hole-transporting ability.^19*a*^ The device where DMTPS-DPA functions as both the light emitter and hole-transporter (ITO/DMTPS-DPA (80 nm)/TPBi (10 nm)/Alq_3_ (30 nm)/LiF (1 nm)/Al (100 nm)) radiates a yellow EL emission at 548 nm (CIE = 0.40, 0.57), shows a low turn-on voltage of 3.1 V, and has a high luminance up to 14 038 cd m^–2^. The maximum current, power and external efficiencies of the device were 7.60 cd A^–1^, 6.94 lm W^–1^ and 2.26%, respectively. These data are close to those attained from the device with an additional NPB hole-transporting layer, demonstrating the good bifunctionality of DMTPS-DPA. The hole-transporting ability of the triphenylamine groups is dominant in DMTPS-DPA, while the intrinsic low-lying LUMO energy level of the silole core facilitates electron injection, and thus, exciton recombination.

## 4. Conclusion and outlook

Siloles are kinds of classic silicon-containing heterocyclic compounds developed several decades ago, which received considerable attention due to their unique σ*–π* conjugated electronic structures. The discovery of the intriguing AIE phenomenon endows siloles with brand new research topics, and they are currently emerging as promising luminogenic materials with diverse applications in frontier research for materials science, environmental protection and biotechnology. So far, remarkable progress towards the development of silole-based materials has been achieved, which enables us to deeply understand the AIE mechanism and structure–property relationship of siloles, and guides us to design and synthesize efficient luminescent materials with specific properties and desired functionalities. To shed light on the substitution effect, herein, the AIE characteristics of siloles containing various substituents such as flexible silyl groups, rod like ethynyl groups, and planar fluorescent chromophores are discussed, and the influence of the conjugation pattern between the substituents and siloles is described as well.

The working mechanism behind the AIE phenomenon of propeller-like siloles is rationalized to be the restriction of IMR process of the aromatic rotors against the silole ring stator. The rotors at the 3,4-positions contribute little to determining the absorption and emission wavelengths, but they are virtually crucial for the AIE activities of siloles, whose rotational motions efficiently deactivate the excited states of the molecules in a nonradiative manner, and thus, lead to weak emissions in the solution states. Without them, the steric congestion of the molecule is reduced, and the conjugation between substituents at the 2,5-positions and the central silole ring is improved, making a better conjugated, more rigid molecular backbone. The emission efficiency in solution is thus enhanced and the AIE effect disappears. The substituents at the 2,5-positions control the effective conjugation lengths and electronic structures of siloles, and thus impact the emission wavelengths and efficiencies remarkably. The good conjugation between the large planar fluorescent groups at the 2,5-positions and the silole ring compensates for the emission quenching effect caused by the rotational motion, and enhances the emission efficiency in solution. However, the emission can be red-shifted and decreased in the aggregated state because large planar groups are prone to forming strong π–π intermolecular interactions. The collective effect of both factors gives rise to no or faint AIE features. The substituents located at the 1-position of the silole ring and those weakly conjugated to the molecular backbone exert no obvious effect on the emission properties of the siloles in solution, but they have apparent impacts on the emission behaviors in the aggregated state *via* intermolecular interaction modulation.

Guided by the AIE mechanism deciphering and the structure–property correlation understanding, desired emission behaviors and functionalities can be readily realized by simple chemical modifications. A large variety of new silole-based functional materials have been developed, with tunable emission colors and good fluorescence efficiencies. The new silole derivatives have shown great potential in chemosensors, biological probes and imaging, chiral supramolecular self-assembly, OLEDs, *etc.* Thanks to the excellent AIE properties, these silole derivatives have advantages in a wide array of high-tech applications. For example, they can be used to fabricate high-performance non-doped OLEDs, and the complicated and troublesome doping technique for alleviating the ACQ problem of many conventional fluorescent and phosphorescent light emitters can be avoided. High solid-state emission efficiencies and good electron-transporting abilities are highly favored in non-doped OLEDs. In bioimaging applications, photobleaching is a common problem with organic luminescent reagents, which is unfavorable for long-term tracking. Fabricating luminescent dyes into small dots can improve the photostability of the materials. However, in most cases, this method will cause the adverse effect of emission quenching. In this regard, AIE-active siloles are perfect candidates for preparing fluorescent dots, in which both high photostability and bright fluorescence can be facilely achieved.

One defect that should be pointed out is that the emission color of most siloles is in the green to yellow region, and efficient blue emission has hardly been achieved yet. Even the archetypal 2,3,4,5-tetraphenylsiloles only give strong green emissions in the aggregated state, which is not as interesting as red emissions for applications in bioscience and OLEDs. Weakening the conjugation of the molecule can move the emission to the blue region, but may cause a decrease in emission efficiency. Efficient red emission is envisioned to be more readily achievable by the endeavors of molecular engineering. In view of this, the development of functional siloles with long-wavelength emissions should be a promising direction for research, which is still in an infant stage.

Current achievements are anticipated to trigger more interest and ideas from researchers and to further promote and broaden applications of AIE-active siloles in various environmental, biological and optoelectronic fields. By structure–property relationship understanding and AIE mechanism deciphering, the AIE studies can extend from peculiar propeller-like siloles to more common fluorescent systems, such as green fluorescent proteins, bacterial luciferase fluorophores, coumarin derivatives, *etc.* In addition to the restriction of intramolecular rotation, restriction of other kinds of intramolecular motions, such as vibration or twisting, may play a role in these new AIE systems. These fluorophores have diverse molecular structures from natural products to artificial compounds containing pure hydrocarbons or heteroatoms or metal ions. Benefiting from the intriguing AIE nature, they have a prosperous future in diverse research frontiers.
